# Holmium vanadate nanoparticles as a biosafe and efficient contrast agent for spectral CT imaging of gastritis and colitis

**DOI:** 10.1093/rb/rbag106

**Published:** 2026-06-02

**Authors:** Wenqian Ru, Chunmei Yang, Xin Zhu, Yanlin Wu, Fei Pei, Haoran Chen, Yuanyuan Ma, Qiuyu Meng, Lu Yang, Yong Xu

**Affiliations:** Department of Endocrinology and Metabolism, The Affiliated Hospital of Southwest Medical University, Sichuan Clinical Research Center for Nephropathy, Metabolic Vascular Disease Key Laboratory of Sichuan Province, and Sichuan-Chongqing Joint Key Laboratory of Metabolic Vascular Diseases, Luzhou, Sichuan 646000, China; Department of Radiology, The Affiliated Hospital, Southwest Medical University, Precision Imaging and Intelligent Analysis Key Laboratory of Luzhou, Luzhou, Sichuan 646000, China; Department of Radiology, The Affiliated Hospital, Southwest Medical University, Precision Imaging and Intelligent Analysis Key Laboratory of Luzhou, Luzhou, Sichuan 646000, China; Department of Radiology, The Affiliated Hospital, Southwest Medical University, Precision Imaging and Intelligent Analysis Key Laboratory of Luzhou, Luzhou, Sichuan 646000, China; Department of Endocrinology and Metabolism, The Affiliated Hospital of Southwest Medical University, Sichuan Clinical Research Center for Nephropathy, Metabolic Vascular Disease Key Laboratory of Sichuan Province, and Sichuan-Chongqing Joint Key Laboratory of Metabolic Vascular Diseases, Luzhou, Sichuan 646000, China; Department of Endocrinology and Metabolism, The Affiliated Hospital of Southwest Medical University, Sichuan Clinical Research Center for Nephropathy, Metabolic Vascular Disease Key Laboratory of Sichuan Province, and Sichuan-Chongqing Joint Key Laboratory of Metabolic Vascular Diseases, Luzhou, Sichuan 646000, China; Department of Endocrinology and Metabolism, The Affiliated Hospital of Southwest Medical University, Sichuan Clinical Research Center for Nephropathy, Metabolic Vascular Disease Key Laboratory of Sichuan Province, and Sichuan-Chongqing Joint Key Laboratory of Metabolic Vascular Diseases, Luzhou, Sichuan 646000, China; Department of Endocrinology and Metabolism, The Affiliated Hospital of Southwest Medical University, Sichuan Clinical Research Center for Nephropathy, Metabolic Vascular Disease Key Laboratory of Sichuan Province, and Sichuan-Chongqing Joint Key Laboratory of Metabolic Vascular Diseases, Luzhou, Sichuan 646000, China; Zhejiang Collaborative Innovation Center for Full-Process Monitoring and Green Governance of Emerging Contaminants, Interdisciplinary Research Academy (IRA), Zhejiang Shuren University, Hangzhou 310015, China; Department of Endocrinology and Metabolism, The Affiliated Hospital of Southwest Medical University, Sichuan Clinical Research Center for Nephropathy, Metabolic Vascular Disease Key Laboratory of Sichuan Province, and Sichuan-Chongqing Joint Key Laboratory of Metabolic Vascular Diseases, Luzhou, Sichuan 646000, China; Department of Endocrinology and Metabolism, The Affiliated Hospital of Southwest Medical University, Sichuan Clinical Research Center for Nephropathy, Metabolic Vascular Disease Key Laboratory of Sichuan Province, and Sichuan-Chongqing Joint Key Laboratory of Metabolic Vascular Diseases, Luzhou, Sichuan 646000, China

**Keywords:** spectral CT imaging, gastritis, colitis, CT contrast agent, clinical translation

## Abstract

The development of a biosafe and high-performance spectral computed tomography (CT) contrast agent for the specific and non-invasive diagnosis of gastrointestinal diseases represents a significant clinical need. Unlike conventional CT, which is susceptible to artifacts from polychromatic X-ray beams, spectral CT distinguishes itself by utilizing material-specific, energy-dependent attenuation data. This capability enables superior differentiation between tissue types, leading to reduced background noise and improved contrast specificity. In this study, we developed hyaluronic acid-functionalized holmium vanadate nanoparticles (HA-HoVO_4_ NPs) for targeted spectral CT imaging of gastritis and colitis. HA-HoVO_4_ NPs exhibited excellent biocompatibility, stability and high X-ray attenuation properties. Following oral administration, HA-HoVO_4_ NPs not only clearly delineated the gastrointestinal tract anatomy but also selectively accumulated in inflammatory regions in murine models of gastritis and colitis, demonstrating excellent targeting capability. The combination of HA-mediated targeting with spectral CT imaging significantly enhanced diagnostic sensitivity and accuracy. The findings indicate that HA-HoVO_4_ NPs represent a highly promising targeted spectral CT contrast agent, offering new possibilities for the precise and non-invasive diagnosis of gastrointestinal inflammatory diseases. This technology thus promises to advance inflammation identification and treatment efficacy evaluation under imaging guidance, thereby providing more reliable support for clinical diagnosis and management.

## Graphical abstract

**Graphical Abstract:**
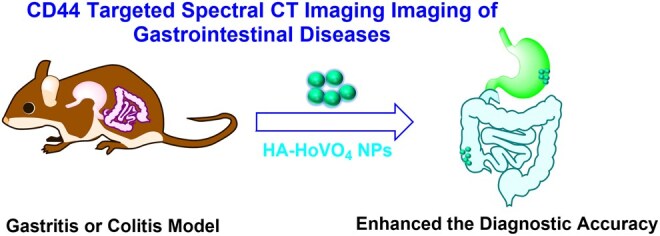

## Introduction

Gastrointestinal diseases, such as gastritis and colitis, represent a major global health burden with a consistently rising incidence [1–3]. The accurate and early diagnosis of these conditions is critically important, as their initial clinical manifestations are often non-specific, leading to high rates of misdiagnosis [4–6]. Left undetected, chronic gastrointestinal inflammation can progress to serious complications including ulceration, bleeding, and an increased risk of gastric or colorectal cancer [7–10]. Currently, several clinical imaging modalities are employed for the diagnosis of gastrointestinal diseases [11–13]. While endoscopy allows direct mucosal visualization, it is invasive and carries risks of complications [14]. Conversely, non-invasive techniques such as X-ray barium meal examination offer limited resolution and sensitivity [15]. Traditional computed tomography (CT), despite its advantages in speed and spatial resolution, is often hindered in gastrointestinal applications by intraluminal content interference and poor soft-tissue contrast, which impedes the clear identification of early mucosal lesions [16]. Therefore, there is a pressing clinical need for advanced, non-invasive diagnostic strategies that can overcome these limitations to enable precise detection and assessment of gastrointestinal diseases.

Spectral CT imaging, as an advanced diagnostic technology, provides high-resolution visualization of the gastrointestinal tract with clear anatomical structures and pathological features [17–20]. By analyzing X-ray attenuation characteristics across different monochromatic energy levels, it further enables quantitative assessment of tissue composition, thereby improving diagnostic accuracy and supporting disease differentiation [5]. As a result, the advancement of spectral CT is crucial for achieving early detection and prognostic evaluation of gastrointestinal diseases. Nonetheless, the inherent limitations of conventional CT contrast agents, such as barium- and iodine-based formulations, hinder their effectiveness in gastrointestinal spectral CT applications [21, 22]. Although barium sulfate delivers strong luminal contrast for morphological outlining, it offers poor soft-tissue differentiation, limiting its sensitivity for early or subtle mucosal lesions. In addition, retained barium carries a risk of intestinal obstruction [23, 24]. Iodinated agents, while improving tissue contrast and lesion conspicuity, have short gastrointestinal transit times that often preclude sustained imaging windows and are associated with potential adverse effects including allergic reactions and nephrotoxicity [25]. Consequently, the development of novel contrast agents that are safe, effective and tailored for spectral CT imaging represents a significant objective.

The rapid progress in nanotechnology has facilitated the development of spectral CT contrast agents. Nanomaterials have distinctive physicochemical properties such as small size, high surface-area-to-volume ratio and facile surface functionalization, demonstrating substantial potential in biomedical applications [26, 27]. Currently, a range of nanomaterials is developed for spectral CT imaging, including those based on lanthanides (e.g. holmium, Ho) [5, 28], gold (Au) [29], bismuth (Bi) [30, 31], erbium (Er) [32] and transition metals (TM) [33–35]. Among them, lanthanide-based nanomaterials have garnered considerable interest owing to their strong X-ray attenuation and K-edge energies that are located within the standard CT imaging energy range (80–120 keV), making them ideal candidates for spectral CT contrast agents. Specifically, the lanthanide element Ho (atomic number Z = 67) exhibits a mass attenuation coefficient of 3.49 cm^2^/g at 100 keV and a K-edge energy of 55.6 keV. It is worth noting that the K-edge of the Ho element falls exactly within the ideal energy window for clinical spectral CT imaging (40–80 keV), offering a significant advantage over other high atomic number (high-Z) elements [36, 37]. Although previously reported Bi-based contrast agents (Bi_2_O_3_ [6], BiOCl [16], Bi-DTPA [25]) possess high atomic numbers, their K-edge value is 90.5 keV, which is above the ideal imaging range and may lead to a low signal-to-noise ratio at clinically relevant energies [38]. In contrast, cerium-based contrast agents (such as dextran-coated CeO_2_) have a K-edge energy of 40.4 keV [9].

Due to its relatively low energy, the signal tends to overlap with that of background tissues, thereby compromising imaging specificity [39]. Although erbium (with a K-edge energy of 57.5 keV) falls within a suitable energy range [32], its complexes exhibit poor dispersibility and gastrointestinal stability in water [40]. Additionally, the ease of functionalization of Ho-based nanoparticles with targeting ligands or therapeutic drugs broadens their applications in targeted imaging and theranostics [41]. Therefore, Ho-based nanomaterials may serve as effective spectral CT contrast agents to improve the diagnosis of gastrointestinal diseases.

Hyaluronic acid (HA), a naturally occurring polysaccharide, is extensively utilized in biomedical applications due to its excellent biocompatibility, low immunogenicity, and biodegradability [42]. In addition, HA serves as a specific ligand for the CD44 receptor, which is significantly overexpressed on the surfaces of macrophages, fibroblasts and other immune cells within inflammatory tissues, such as those present in gastritis and colitis [43]. This inherent targeting capability allows HA-modified nanoparticles to selectively accumulate at disease sites, enhancing the local concentration of HA-coated contrast agents at gastrointestinal inflammatory lesions. This active targeting strategy is expected to markedly improve the signal-to-noise ratio of spectral CT imaging, leading to superior diagnostic accuracy for gastrointestinal diseases.

**Figure 1:**
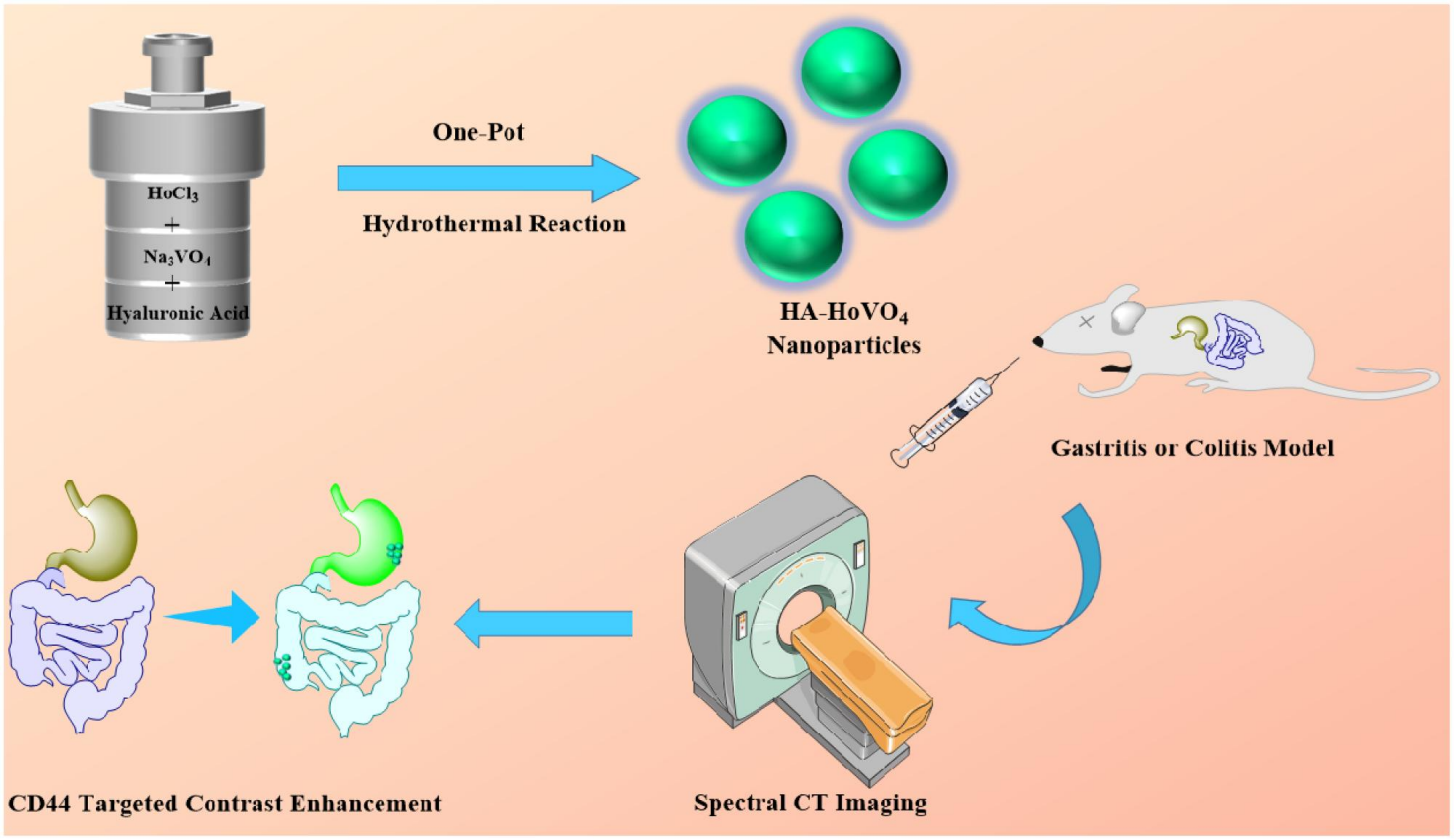
Schematic illustration of HA-HoVO_4_ NPs for spectral CT imaging of the gastrointestinal tract and targeted detection of gastrointestinal inflammatory diseases.

Based on the above analysis, we propose the core hypothesis of this study: surface functionalization of Ho vanadate nanoparticles with HA can construct a novel nanoscale contrast agent that combines good biosafety with efficient targeting capability. However, most of the currently reported Bi-based, CeO_2_-based and Er-based nanomaterials are limited to passive imaging and lack active targeting capability. After oral administration, this contrast agent can specifically accumulate in gastrointestinal inflammatory regions, thereby enabling sensitive detection and accurate delineation of lesions through spectral CT imaging. As shown in Figure 1, to validate this hypothesis, this study successfully synthesized and systematically characterized HA-HoVO_4_ NPs. These nanoparticles were successfully synthesized and demonstrated strong X-ray attenuation capabilities in both *in vitro* and *in vivo* experiments. Upon oral administration, HA-HoVO_4_ NPs enabled clear delineation of gastrointestinal contours and intestinal loop structures. Furthermore, the diagnostic utility of HA-HoVO_4_ NPs was systematically evaluated in rodent models of gastritis and colitis, confirming its ability to support sensitive detection and characterization of inflammatory gastrointestinal diseases using spectral CT imaging.

## Materials and methods

### Regents and materials

All chemicals were of analytical grade and used directly without further purification. Ho (III) chloride hexahydrate (HoCl_3_·6H_2_O, 99.9%) and trisodium tetraoxovanadate dodecahydrate (Na_3_VO_4_·12H_2_O, 99%) were purchased from Macklin (Shanghai, China). HA (sodium salt, molecular weight (MW) ≈ 1500–1800 kDa) and lipopolysaccharides (LPS) were obtained from Sigma-Aldrich (St. Louis, MO, USA). Indomethacin was procured from Acros (Beijing, China). Dextran sodium sulfate (DSS, MW ≈360000–500000 Da) was supplied by MP Biomedicals (Irvine, USA).

### Synthesis of HA-HoVO_4_ NPs

HA-HoVO_4_ NPs were synthesized via a minor modification of the literature protocol [44]. In a typical procedure, HoCl_3_·6H_2_O (1 mmol, 0.379 g) and HA (800 mg) were dissolved in 20 mL of deionized water under vigorous stirring. Subsequently, Na_3_VO_4_·12H_2_O (1 mmol, 0.4 g) was added to the above solution, which was stirred for an additional 30 min. The solution was then transferred into a 50 mL Teflon-lined stainless-steel autoclave and heated at 140°C for 6 h. After completion, the system was allowed to cool naturally to room temperature. The obtained product was collected by centrifugation, washed three times with deionized water, and stored at 4°C for further use.

### Characterization

The morphology of the HA-HoVO_4_ NPs was characterized using transmission electron microscopy (TEM) on a Tecnai G2F20 instrument (FEI Ltd., USA) at an accelerating voltage of 200 kV. Fourier-transform infrared (FT-IR) spectroscopy was performed on an IR Affinity-1S spectrometer (Shimadzu, Japan) using the potassium bromide (KBr) pellet technique. X-ray diffraction (XRD) analysis was conducted on a Bruker D8 Advance X-ray diffractometer (Germany) to determine the crystal structure of the HA-HoVO_4_ NPs. The concentrations of Ho and vanadium element (V) in HA-HoVO_4_ NPs were quantified by inductively coupled plasma optical emission spectroscopy (ICP-OES) on ThermoICPOES7200 (ThermoFisher, USA). Surface composition and chemical states were determined by X-ray photoelectron spectroscopy (XPS, Thermo Escalab 250Xi). The hydrodynamic diameter was obtained from dynamic light scattering (DLS) measurements performed on a Zetasizer Nano ZS90 (Malvern Instrument, Worcs, UK) at 25°C. Particle size was calculated using the Zetasizer Ver. 8.01.4906 (Serial Number: MAL1059346) with the cumulant method for calculating the z-average hydrodynamic diameter and the polydispersity index (PDI).

### *In vitro* CT imaging

To assess CT imaging capabilities, solutions of HA-HoVO_4_ and iohexol at various concentrations (0, 5, 10, 20 and 40 mM Ho or I) were prepared. On CT transverse images, three consecutive central slices of each sample were selected. An elliptical Region of Interest (ROI) covering approximately 80% of the sample cross-sectional area was manually delineated to minimize partial volume effects. The mean HU value was measured for each slice, and the results were presented as the mean ± standard deviation (SD) of the three slices. Spectral CT imaging was performed using an IQON spectral CT system (Philips, Netherlands). The scanning parameters were set as follows: field of view of 150 × 150 mm, slice thickness of 0.4 mm, tube current of 100 mA, tube voltage of 120 kV. All samples were placed at consistent positions to ensure identical irradiation conditions across samples, thereby avoiding experimental variability caused by differences in irradiation conditions. Virtual monochromatic images were obtained at 10 keV intervals across the photon energy range of 40–140 keV. Three-dimensional (3D) reconstructions were generated using the Philips Intellispace Portal workstation. The CT values of each sample were measured using image analysis software and plotted as a function of concentration.

### The solubility and stability of HA-HoVO_4_ NPs

HA-HoVO_4_ NPs were dispersed in different media at 37°C for 7 days, including 0.9% NaCl solution, phosphate buffer saline (PBS), Roswell Park Memorial Institute-1640 medium (RPMI-1640), Dulbecco’s modified Eagle medium (DMEM) and fetal bovine serum (FBS). Photos were collected at 7 and 14 days to evaluate the colloidal stability.

### Cytotoxicity assessment

RAW264.7 (mouse monocyte macrophage leukemia cell line), MCF-10A (human normal mammary epithelial cells) and CT26 (mouse colon cancer cells) were cultured in DMEM or RPMI-1640 medium supplemented with 10% FBS and 1% penicillin-streptomycin under 5% CO_2_. The cytotoxicity of HA-HoVO_4_ NPs was detected by the cell counting kit-8 (CCK-8) assay. Cells were seeded in 96-well plates at a density of 1 × 10^4^ cells/well and incubated for 24 h. Subsequently, the cells were treated with HA-HoVO_4_ NPs (0–200 µg/mL) for another 24 h. After treatment, the cells were washed with PBS. Next, 100 μL of fresh medium and 10 μL of CCK-8 reagent were added to each well. Following 2 h of incubation, the absorbance at 450 nm was measured using a microplate reader, and cell viability was calculated accordingly.

### Hemocompatibility assay

Fresh mouse blood (1 mL) was collected in an anticoagulant tube, diluted with PBS, and allowed to stand for 1 h. After centrifugation, the erythrocyte pellet was washed three times with PBS and resuspended in PBS. The erythrocyte suspension was then incubated with HA-HoVO_4_ NPs at concentrations of 50, 100, 200 and 400 μg/mL. PBS and pure water were used as negative and positive controls, respectively. After 2 h of incubation, the mixtures were centrifuged, and hemolysis was preliminarily assessed by visual inspection. The supernatant from each sample was transferred to a 96-well plate, and the absorbance at 540 nm was measured using a microplate reader. The hemolysis rate was calculated as follows:


$$\mathrm{Hemolysis}\;\mathrm{rate}(\%)=(A_{s}-A_{n})/(A_{p}-A_{n})\times 100\%,$$

where *A*_S_, *A*_n_ and *A*_p_ represent the absorbance of the sample, negative control and positive control, respectively.

### *In vivo* biodistribution

All procedures and animal experiments were approved by the ethics and scientific committee of the animal care and treatment committee of the Southwestern Medical University (Project Number: 20231011-001). The *in vivo* biodistribution of HA-HoVO_4_ NPs was evaluated after oral administration in mice (200 μL, 144 mg Ho/kg body weight). Animals were euthanized at 24 h post-gavage, and major organs (heart, liver, spleen, lungs and kidneys) were collected. In addition, to investigate the clearance pathway, gastrointestinal tissues (stomach, small intestine and colon) were collected at 0, 4 and 28 h post-administration. The Ho content in each tissue was quantified using ICP-OES. Mice administered with 5% glucose solution were used as a control.

### Cellular targeting assay

To evaluate the inflammatory targeting ability of HA-HoVO_4_ NPs, RAW 264.7 cells were stimulated with 1 μg/mL LPS at 37°C for 24 h to establish an inflammatory model, and unstimulated cells served as the control. Both groups were then treated with HA-HoVO_4_ NPs or HoVO_4_ NPs at concentrations of 0, 100, 200 and 400 μg/mL. In competitive binding experiments, free HA (5 mg/mL) was pre-incubated with cells for 1 h to block CD44 receptors prior to nanoparticle treatment. After 4 h of incubation with the nanoparticles, cells were washed with PBS, detached using trypsin, and prepared for spectral CT imaging.

### CD44 receptor expression analysis

The expression level of cell surface CD44 was detected using an anti-CD44 monoclonal antibody (Saiweiye Biotechnology Co., Ltd., Wuhan, China). The experiment included three groups: LPS-treated RAW264.7 cells, untreated (without LPS stimulation) RAW264.7 cells, and a negative control group. LPS-treated and untreated RAW264.7 cells were washed twice with PBS and then incubated with the anti-CD44 antibody for 30 min at 4°C in the dark. The control group was subjected to the same conditions but without staining with the anti-CD44 antibody. After incubation, the cells were washed three times, centrifuged, resuspended, and analyzed using a flow cytometer (FACS Aria).

### *In vivo* biosafety evaluation

To assess the *in vivo* biosafety of HA-HoVO_4_ NPs, BALB/c mice (6 weeks old, 18–20 g) were acclimatized for 7 days and then randomly divided into a healthy control group and a treated group (*n* = 4). The treated group received HA-HoVO_4_ NPs via oral gavage (200 μL, 144 mg Ho/kg body weight), while the control group received an equivalent volume of normal saline. Mice were euthanized on day 1 and day 14 post-administration. Histopathological examination and blood biochemical analysis were performed to evaluate systemic toxicity. Major organs, including the heart, liver, spleen, lungs, kidneys, stomach, small intestine and large intestine, were collected and fixed in 4% paraformaldehyde for hematoxylin and eosin (H&E) staining. Blood samples were collected from each mouse, and serum was analyzed for the following parameters: Liver function markers: alanine aminotransferase (ALT), aspartate aminotransferase (AST), alkaline phosphatase (ALP), albumin (ALB), total protein (TP) and globulin (GLO). Kidney function markers: creatinine (CREA), uric acid (UA) and urea (UREA). Body weights were recorded daily throughout the 14-day observation period.

### *In vivo* CT imaging of gastritis

An acute gastritis rat model was established based on a published protocol using oral administration of alcohol and aspirin [4]. At 4 h post modeling, rats received 200 μL of HA-HoVO_4_ NPs (144 mg Ho/kg) or iohexol (112 mg I/kg) orally. CT imaging was performed at the following time points: before administration (pre) and at 5 min, 30 min, 1 h, 4 h, 8 h, 12 h and 24 h post-administration, using the same scanning parameters as described previously. Normal rats underwent the same dosing and imaging procedures as controls. The temporal changes in gastric CT images were compared between gastritis and normal rats to evaluate the performance of HA-HoVO_4_ NPs and iohexol in gastritis imaging.

### *In vivo* CT imaging of colitis

An acute colitis model was induced using DSS. Female BALB/c mice were administered 5% DSS in drinking water for 6 days, followed by regular water; healthy control mice received water only. Body weight was recorded daily throughout the study. To validate successful modeling, colon tissues were collected (*n* = 6) for measurement of colon length and fixed in 4% paraformaldehyde for H&E staining.

DSS-induced colitis mice and healthy mice were orally administered iohexol (*n* = 5) or HA-HoVO_4_ NPs (*n* = 5) at doses of 112 mg I/kg or 144 mg Ho/kg body weight, respectively. CT imaging was performed at multiple time points (pre-administration, 5 min, 30 min, 1 h, 2 h, 4 h, 8 h, 12 h, and 28 h post-administration) using a tube voltage of 120 kV. Scanning parameters included a field of view of 150 × 150 mm, a slice thickness of 0.4 mm and a tube current of 100 mA. Healthy and colitis mice receiving equivalent doses of HA-HoVO_4_ NPs or iohexol served as controls and were imaged under identical conditions. Spectral CT imaging was conducted under different monochromatic energy levels; 3D reconstruction views, and coronal CT images were acquired by post-processing workstation. Three consecutive slices were selected from the central region of colitis on cross-sectional CT images, and then ROIs were placed in the central area of the inflammatory site to obtain their mean HU values. Additionally, large intestine tissue samples from DSS-induced colitis mice were collected 28 h after HA-HoVO_4_ NPs administration for electron microscopy analysis.

### Statistical analysis

Continuous variables were expressed as mean ± SD when the distribution of data was normal, or as median when it was outside the bounds of normality. The variables were compared using independent *t*-tests or Wilcoxon rank sum tests, when appropriate. Significance levels were defined as follows: *P < 0.05, **P < 0.01, ***P < 0.001, ***P < 0.0001; “ns” indicated no significant difference. All statistical analyses were performed using GraphPad Prism software.

## Results

### Synthesis and characterization of HA-HoVO_4_ NPs

**Figure 2:**
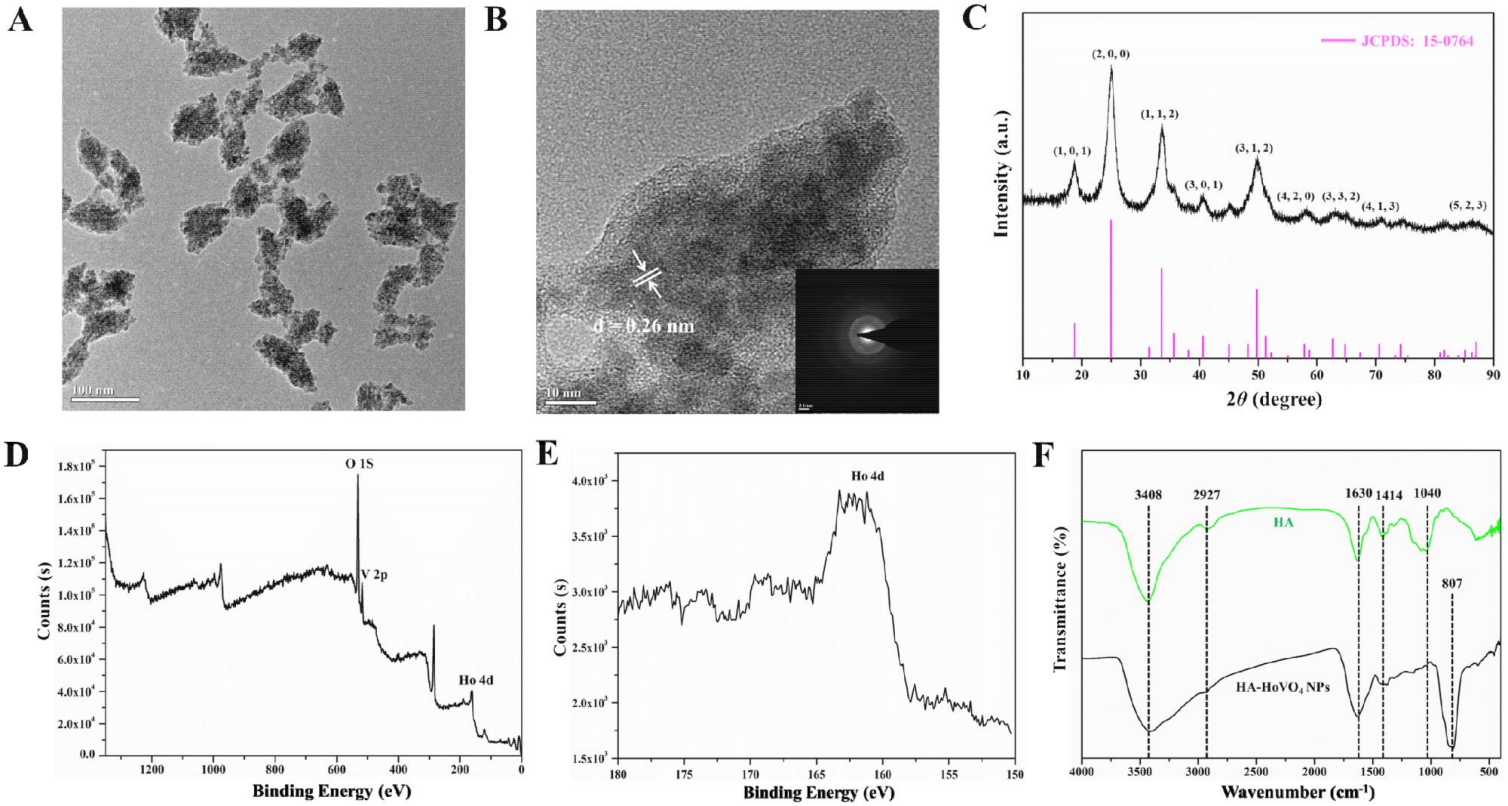
Characterization of HA-HoVO_4_ NPs. (**A**) TEM image. (**B**) HR-TEM image (inset: SAED pattern). (**C**) XRD spectrum of HA-HoVO_4_ compared with the standard JCPDS file of HoVO_4_ (card no. 15-0764). (**D**) XPS spectrum of HA-HoVO_4_ NPs. (**E**) High-resolution XPS spectra of Ho 4d. (**F**) FT-IR spectra of HA-HoVO_4_ NPs and HA.

The CD44-targeting HA-HoVO_4_ NPs were achieved via a hydrothermal method, and HA as targeting molecules was capped on the surface of HoVO_4_ NPs. As shown in Figure 2A and B, HA-HoVO_4_ NPs exhibited a uniform spherical morphology. High-resolution TEM (HR-TEM) images revealed lattice fringes with an interplanar spacing of 0.26 nm, and the corresponding selected area electron diffraction (SAED) pattern displayed continuous rings, confirming the crystalline nature of the nanoparticles (inset in Figure 2B). DLS measurements indicated a hydrodynamic diameter of 165.7 ± 1.6 nm (Figure S1); the corresponding PDI was 0.048 ± 0.030, and the surface zeta potential was −18.9 ± 0.6 mV (Figure S2), suggesting that HA-HoVO_4_ NPs may have good colloidal stability attributable to the HA coating. Energy dispersive spectroscopy confirmed the presence and homogeneous distribution of Ho, V and O within the HA-HoVO_4_ NPs (Figure S3).

Furthermore, as illustrated in Figure 2C, XRD analysis confirmed that HA-HoVO_4_ NPs exhibited a typical monoclinic crystal structure, with all diffraction peaks consistent with the standard pattern of HoVO_4_ (JCPDS No. 15-0764). XPS analysis and high-resolution profile of Ho 4d, V 2p and O 1s further verified the presence and chemical states of Ho, V and O in the nanoparticles (Figure 2D and E). FT-IR spectroscopy revealed noticeable shifts in characteristic HA absorption bands (O–H, C–H, C–O and C=O), indicating successful surface functionalization with HA (Figure 2F). Then thermogravimetric analysis was used to analyze the weight loss of HA-HoVO_4_ NPs, which confirmed a 61.27% loading of HoVO_4_ in the nanoparticles (Figure S4). Quantitative analysis by ICP-OES showed an atomic Ho:V ratio close to 1:1, and the yield was as high as 94%. Together, these results confirm the successful synthesis and structural integrity of HA-HoVO_4_ NPs.

### *In vitro* CT imaging

**Figure 3:**
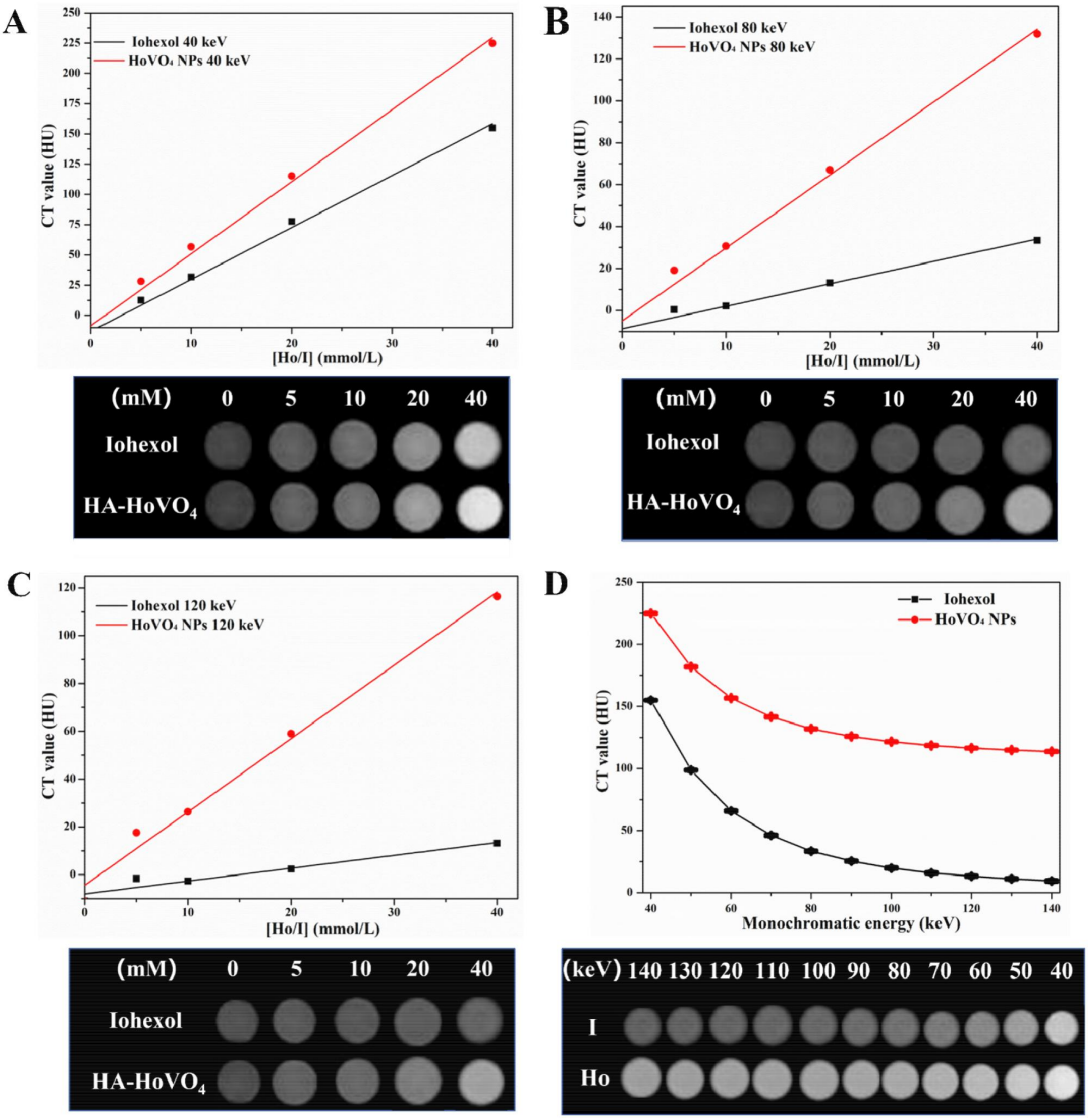
Spectral CT imaging performance of HA-HoVO_4_ NPs and iohexol. HU curves and spectral CT images of HA-HoVO_4_ NPs and iohexol at concentrations of 0, 5, 10, 20 and 40 mM (Ho or I) acquired at (**A**) 40 keV, (**B**) 80 keV and (**C**) 120 keV. (**D**) HU values and corresponding images of HA-HoVO_4_ NPs and iohexol (40 mM) across different monochromatic energies.

To detect the CT imaging performance of HA-HoVO_4_ NPs, the X-ray absorption capacity of HA-HoVO_4_ NPs and iohexol at different tube voltages (40–140 kV) was studied by spectral CT. At increasing concentrations, HA‑HoVO_4_ NPs produced brighter CT images and higher CT values than iohexol, and both agents exhibited a linear concentration-dependent increase in CT values (Figure 3A–C). At increasing monochromatic energy levels from 40 to 120 keV, the difference in attenuation values between HA‑HoVO_4_ NPs and iohexol became more pronounced. At equivalent elemental concentrations, HA‑HoVO_4_ NPs consistently yielded higher image brightness compared to iohexol across the voltage range of 40–140 keV (Figure 3D), indicating that HA‑HoVO_4_ NPs enabled high‑quality imaging at lower doses, which may help reduce potential side effects by minimizing the required dosage. In clinical gastrointestinal spectral CT imaging, the commonly used energy range is 40–80 keV, within which HA-HoVO_4_ NPs exhibited higher HU values than iodine-based contrast agents, benefiting from their higher atomic number and K-edge effect. Taken together, these results provided evidence of the superior X‑ray attenuation properties of HA‑HoVO_4_ NPs and supported their strong potential for spectral CT imaging.

### *In vitro* and *in vivo* biocompatibility assessment

The stability of HA‑HoVO_4_ NPs was evaluated in various physiologically relevant media, including 0.9% NaCl solution, PBS, RPMI-1640, DMEM and FBS. No visible precipitation or significant aggregation was observed after 7 or 14 days of incubation, confirming the excellent colloidal stability and structural integrity of the nanoparticles (Figure S5). Their excellent colloidal stability is further demonstrated by the similar hydrodynamic size and apparent zeta potential after being dissolved in different media (H_2_O, PBS and 0.9% NaCl solution) (Figure S6).

**Figure 4:**
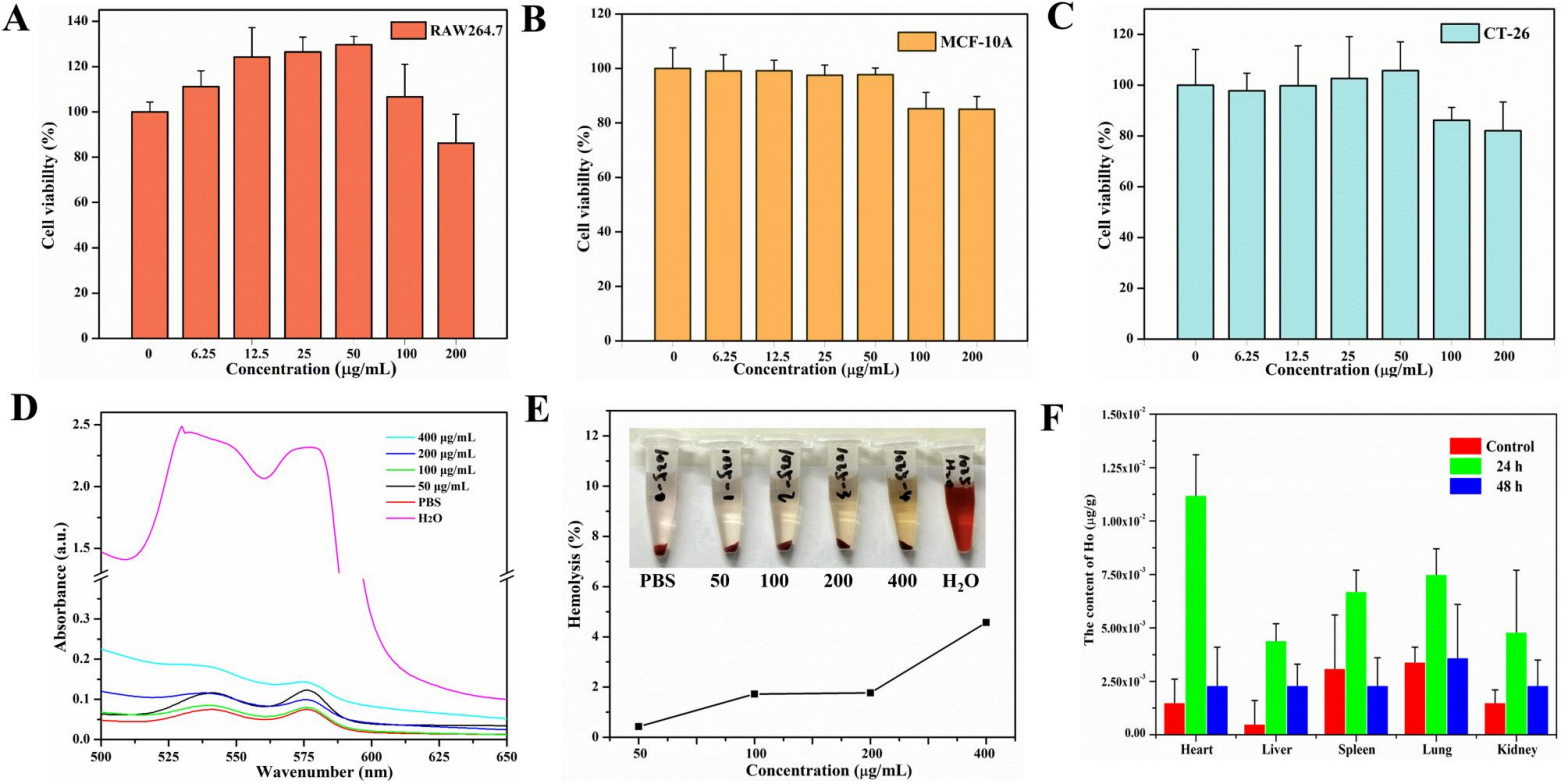
Biocompatibility of HA-HoVO_4_ NPs. (**A–C**) Viability of RAW264.7, MCF-10A and CT26 cells after treating with HA-HoVO_4_ NPs (0, 6.25, 12.5, 25, 50, 100 and 200 μg/mL) for 24 h (*n* = 3). (**D**) Ultraviolet-visible absorption spectra of hemoglobin in the supernatant of HA-HoVO_4_ NPs and RBC mixture. (**E**) Hemolysis rate of HA-HoVO_4_ NPs at different concentrations (50, 100, 200 and 400 μg/mL). (**F**) Biodistribution of Ho in major organs of mice at different time points following oral administration of HA-HoVO_4_ NPs (*n* = 3).

Prior to *in vivo* CT imaging, the biocompatibility of HA‑HoVO_4_ NPs was systematically assessed *in vitro* and *in vivo*. Cytotoxicity was evaluated in RAW264.7, MCF-10A and CT26 cell lines using the CCK-8 assay. After 24 h of exposure to HA‑HoVO_4_ NPs at concentrations ranging from 0 to 200 μg/mL, cell viability remained high across all cell types (Figure 4A–C), demonstrating the low cytotoxicity of the nanoparticles.

Hemocompatibility assessment revealed negligible hemolytic activity for HA-HoVO_4_ NPs even at a concentration of 400 μg/mL (Figure 4D). Across the tested concentration range, the hemolysis rate remained extremely low (Figure 4E), confirming the favorable blood compatibility of the nanoparticles. To evaluate the biodistribution and clearance profile of HA-HoVO_4_ NPs, Ho content in major organs was quantified by ICP-OES in healthy mice following oral administration. As shown in Figure 4F, no significant accumulation of Ho was detected in the heart, liver, spleen, lung or kidney at 24 or 48 h post-administration compared to the control group, indicating limited systemic distribution and efficient clearance, which are advantageous for clinical translation.

A slight increase in Ho content was detected in the lungs at 24 h after oral administration, which returned to the control level by 48 h. We attribute this minimal and transient signal to a small amount of nanoparticles or their dissociated ions being absorbed from the intestine into the systemic circulation, followed by transient physical entrapment in the pulmonary capillary network [45, 46]. These materials were subsequently cleared within 48 h through the body’s clearance mechanisms, such as mucociliary clearance, macrophage phagocytosis and transport, or metabolic excretion [47, 48]. The Ho content at 24 h was extremely low, well below the threshold likely to induce biological effects, and histopathological evaluation of lung tissues at this time point revealed no signs of inflammation or structural abnormalities. Therefore, we consider this phenomenon a normal transient process in nanomaterial biodistribution that does not compromise the *in vivo* biocompatibility of HA-HoVO_4_ NPs. To further elucidate the clearance pathway, Ho content in the gastrointestinal tract (stomach, small intestine and colon) was assessed at 0, 4 and 28 h post-administration. As shown in Figure S7, at 4 h, a high Ho signal was detected in the stomach and small intestine due to oral administration, whereas the signal decreased significantly by 28 h, with residual signal primarily localized in the colon. This time-dependent intestinal transit pattern suggested that the nanoparticles were primarily cleared via the fecal route. Consistent with the biodistribution data, no significant accumulation was observed in major organs at any time point, further confirming the absence of systemic absorption and supporting the favorable biosafety profile of HA-HoVO_4_ NPs.

**Figure 5:**
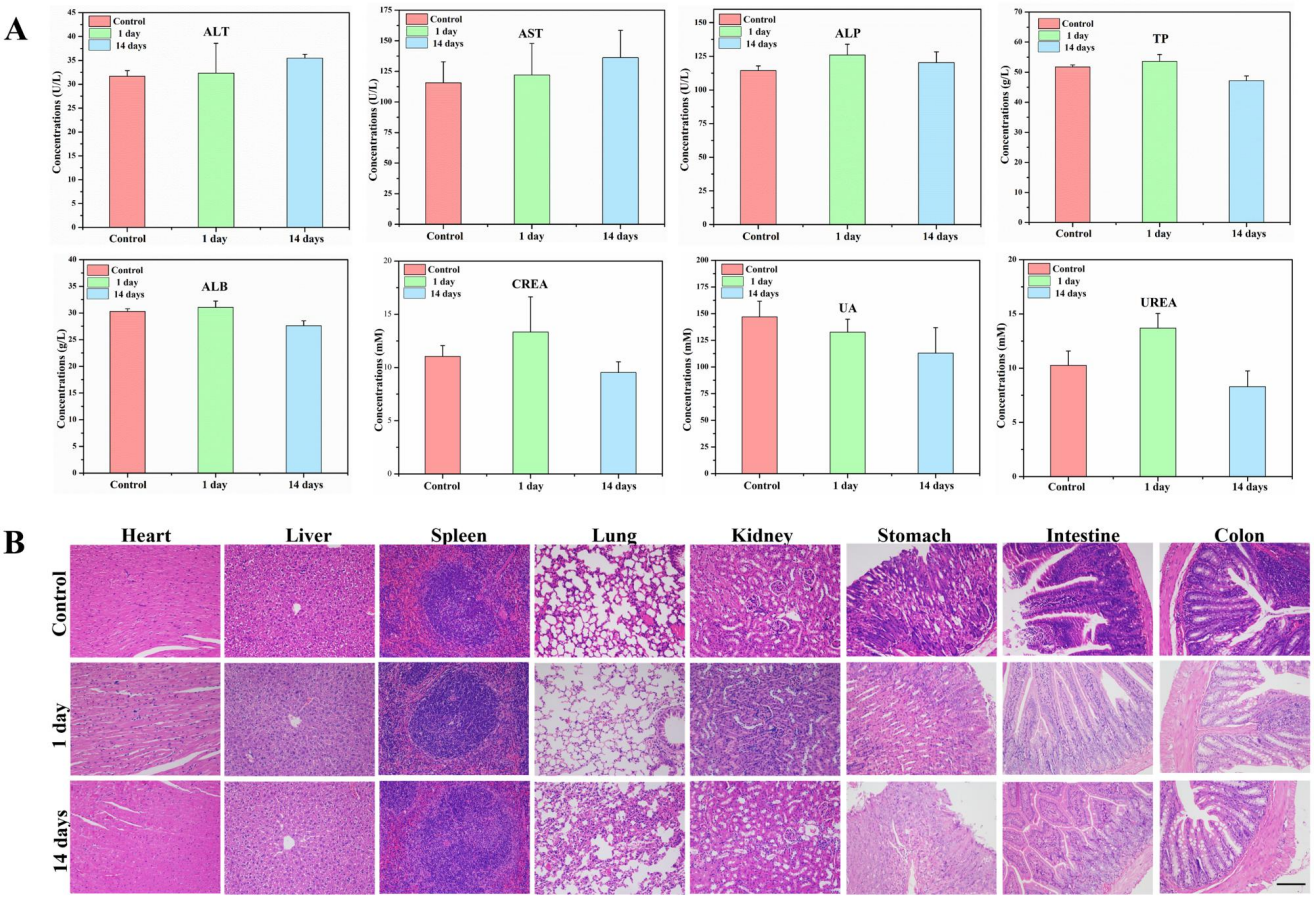
(**A**) Biochemical analysis of mice at 0, 1 and 14 days, including liver and kidney function (*n* = 4). (**B**) H&E staining of major organs (heart, liver, spleen, lung, kidney, stomach, small intestine and large intestine) after oral administration of HA-HoVO_4_ NPs for 0, 1 and 14 days. Scale: 100 μm.

Subsequently, histopathological and biochemical analyses were performed to further assess the biocompatibility of HA-HoVO_4_ NPs. Blood biochemical parameters, including liver function markers (ALT, AST, ALP, ALB, TP, GLO) and kidney function markers (CREA, UA, UREA), showed no significant differences between the HA-HoVO_4_ NP-treated group and the control group at 1 and 14 days post-administration (Figure 5A). Throughout the observation period, the body weight of mice receiving HA-HoVO_4_ NPs increased normally, with no significant deviation from the control group (Figure S8). In addition, histological examination by H&E staining revealed no structural abnormalities, necrosis or inflammatory lesions in major organs (heart, liver, spleen, lung, kidney, stomach, small intestine and large intestine) in HA-HoVO_4_ NP-treated group compared to the control (Figure 5B). These results collectively confirm the favorable *in vivo* biocompatibility of HA-HoVO_4_ NPs.

### *In vitro* targeting assay

**Figure 6:**
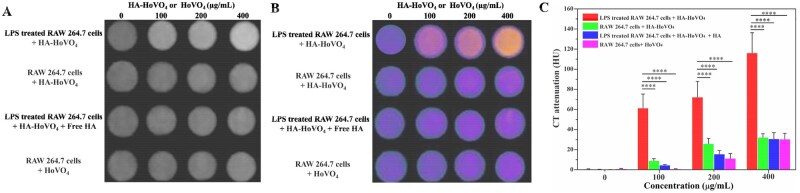
(**A and B**) Representative CT images and (**C**) Quantitative CT values of LPS-treated and untreated RAW 264.7 cells following incubation with HA-HoVO_4_ NPs (*n* = 3). ****P < 0.0001.

To verify the inflammation-targeting ability of HA-HoVO_4_ NPs, RAW 264.7 cells were stimulated with LPS for 24 h to induce cell inflammation. We used flow cytometry to detect the expression level of CD44. The results showed that compared with the untreated group, the surface CD44 expression was significantly increased in the LPS-treated group, indicating that LPS stimulation successfully upregulated CD44 expression (Figure S9). This provides a reliable model basis for subsequent competitive inhibition experiments with free HA. Compared with untreated controls, LPS-induced inflammatory cells showed significantly enhanced uptake of HA-HoVO_4_ NPs (Figure 6A and B). CT analysis further confirmed a marked increase in the CT signal of LPS-treated cells after incubation with HA-HoVO_4_ NPs (Figure 6C), indicating that the nanoparticles possessed strong targeting affinity toward inflammatory cells. To determine whether this targeting behavior depended on the CD44 binding activity of HA, competitive binding assays were performed using excess free HA (5 mg/mL) to block the interaction between HA-HoVO_4_ NPs and CD44 receptors. The CT signal was significantly reduced upon pre-incubation with free HA, confirming that cellular accumulation of HA-HoVO_4_ NPs was mediated through specific CD44 receptor interactions.

### *In vivo* gastrointestinal CT imaging in healthy mice

**Figure 7:**
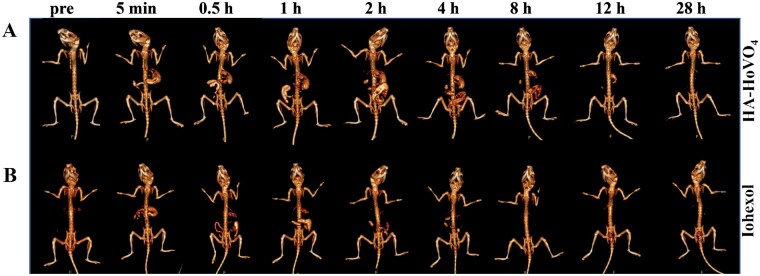
*In vivo* CT imaging of the gastrointestinal tract in healthy mice at different time points after oral administration of (**A)** HA-HoVO_4_ NPs (144 mg Ho/kg) and (**B**) iohexol (112 mg I/kg).

We next evaluated the feasibility of HA-HoVO_4_ NPs in the imaging of the upper and lower gastrointestinal tracts. Healthy mice were orally administered 200 μL of HA-HoVO_4_ NPs (144 mg Ho/kg) or iohexol (112 mg I/kg), followed by CT imaging at multiple time points (Figure 7). Within 5 min after administration of HA-HoVO_4_ NPs, contrast filling was observed in the stomach, duodenum and proximal jejunum. By 2 h, gastric signals diminished while the contours and morphology of small intestinal loops were clearly delineated. After 12 h, most of the nanoparticles had been excreted from the upper gastrointestinal tract, and complete clearance was achieved by 28 h (Figure 7A). HA-HoVO_4_ NPs provided clear and continuous visualization of gastrointestinal anatomy throughout the imaging period. In contrast, iohexol, owing to its low viscosity and poor mucosal adhesion, was rapidly cleared, resulting in fragmented and discontinuous gastrointestinal imaging that was markedly inferior to HA-HoVO_4_ NPs (Figure 7B).

**Figure 8:**
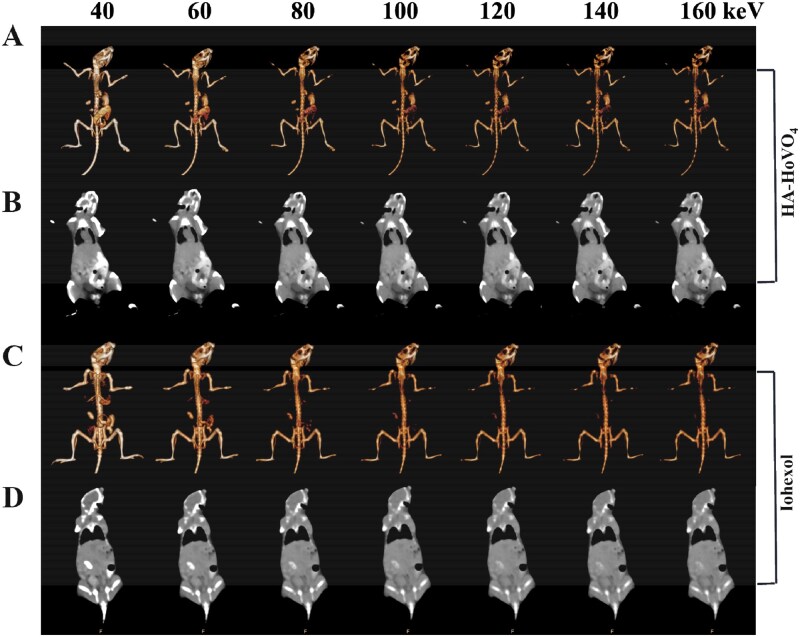
*In vivo* spectral CT imaging in the gastrointestinal tract of normal mice 2 h after oral administration of 200 μL of HA-HoVO_4_ NPs (144 mg Ho/kg) or iohexol (112 mg I/kg), shown as 3D reconstruction views (**A and C**) and coronal CT images (**B and D**) acquired at different monochromatic energies.

To evaluate the *in vivo* spectral CT imaging performance of HA-HoVO_4_ NPs, normal mice were subjected to spectral CT scanning 2 h after oral administration of HA-HoVO_4_ NPs or iohexol. As shown in Figure 8, 3D reconstruction and coronal CT images were acquired across a range of monochromatic energy levels (40–160 keV). At low energy levels (≤60 keV), both contrast agents produced strong signals in the gastrointestinal tract. However, at higher energies (80–160 keV), HA-HoVO_4_ NPs maintained significantly higher tissue contrast, whereas the enhancement from iohexol became nearly undetectable due to its low signal-to-noise ratio. In addition, we performed a systematic quantitative analysis of the raw *in vivo* CT images and calculated the contrast-to-noise ratio (CNR) (Figure S10). The results showed that the CNR value of HA-HoVO_4_ NPs was significantly higher than that of iohexol, particularly at lower energy levels, objectively confirming their superior imaging performance. These results demonstrated that HA-HoVO_4_ NPs offered superior X-ray attenuation and contrast enhancement in spectral CT imaging, highlighting their potential as an effective contrast agent for spectral CT applications.

### *In vivo* CT imaging of gastritis

**Figure 9:**
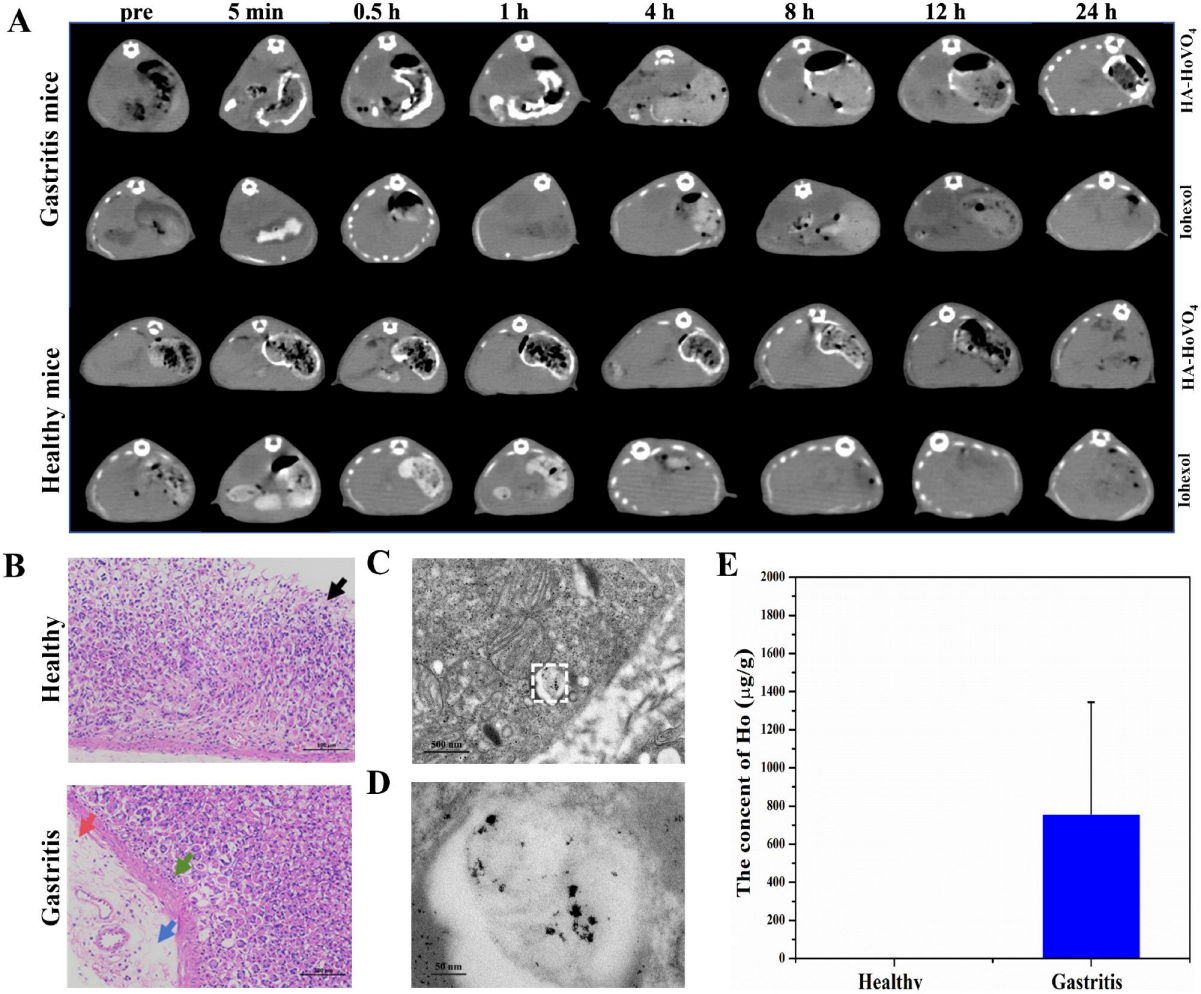
*In vivo* CT imaging of healthy and aspirin-induced acute gastritis rats after administration of HA-HoVO_4_ NPs and iohexol. (**A**) *In vivo* CT images of gastritis and healthy rats at different time points after intragastric administration of HA-HoVO_4_ NPs (144 mg Ho/kg) or iohexol (112 mg I/kg). (**B**) H&E staining of gastric tissue sections from healthy and gastritis groups (scale bar: 100 μm). (**C**) Representative TEM images showing HA-HoVO_4_ NPs accumulation in the stomach of gastritis rats at 24 h. (**D**) Magnified view of the region outlined by the white square in (**C**). (**E**) Ho content in stomach tissues of healthy and gastritis rats 24 h after HA-HoVO_4_ NPs administration, quantified by ICP-OES (*n* = 3).

To assess the performance of HA-HoVO_4_ NPs for gastritis imaging, an acute gastritis model was established in rats using oral administration of alcohol and aspirin. HA-HoVO_4_ NPs or iohexol (1 mL, 60 mg/mL) was administered 4 h post-induction, and CT imaging was performed at multiple time points. As shown in Figure 9A, 5 min after administration of HA-HoVO_4_ NPs in gastritis rats, gastric filling was observed, and the gastric wall became increasingly delineated. By 1 h, the inflamed regions of the gastric wall exhibited markedly enhanced CT signals, allowing clear differentiation from healthy tissue and enabling visualization of inflammation boundaries and extent. In contrast, iohexol provided weak signals at the inflammation site, offering poor differentiation between inflamed and normal tissue.

H&E staining of gastric sections revealed distinct pathological differences between the groups (Figure 9B). The healthy group exhibited intact mucosal architecture with well-arranged cells, whereas the gastritis group showed extensive mucosal erosion (black arrows), structural disruption with necrotic debris, granulocytic infiltration in deep mucosal layers (green arrows), submucosal edema (blue arrows), connective tissue loosening and scattered lymphocytic infiltration (red arrows). Electron microscopy further revealed substantial nanoparticle deposition in gastric parietal cells and intercellular spaces of gastritis rats (Figure 9C and D). Quantitative ICP-OES analysis confirmed significantly higher Ho content in the stomachs of gastritis rats compared to healthy controls 24 h after HA-HoVO_4_ NPs administration, indicating specific accumulation of HA-HoVO_4_ NPs at inflammatory sites (Figure 9E). The Ho element content of HA-HoVO_4_ NPs in the stomach tissue of gastritis rats was also markedly higher than that of HoVO_4_ NPs (Figure S11). These results confirmed the targeted accumulation of HA-HoVO_4_ NPs at inflamed regions and highlighted their potential for precise gastritis imaging.

### *In vivo* CT imaging of colitis

**Figure 10:**
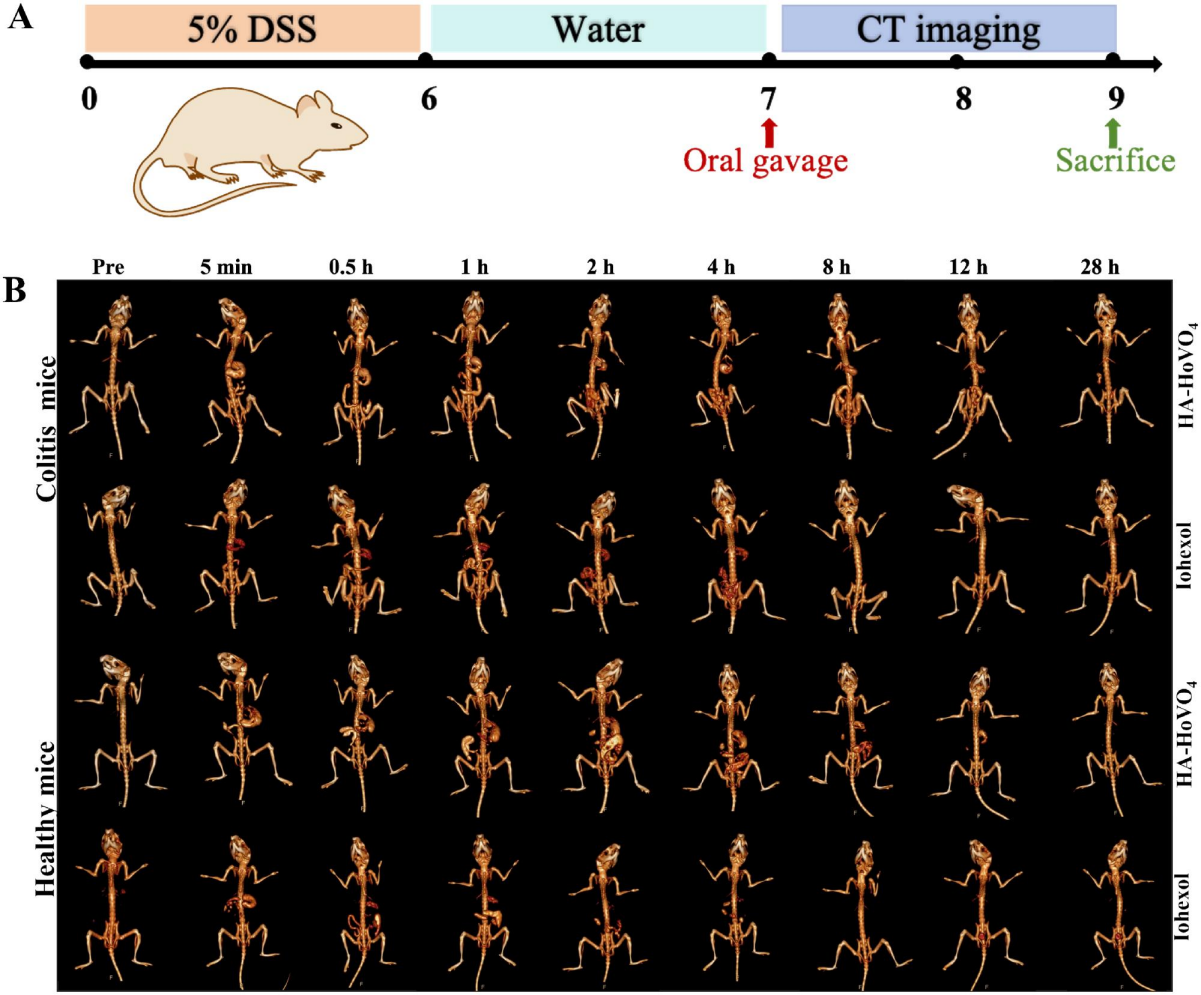
*In vivo* CT imaging of HA-HoVO_4_ NPs in healthy and DSS-induced colitis mice. (**A**) Experimental timeline for colitis induction and CT imaging. (**B**) Representative CT images of colitis and healthy mice before and after oral administration of HA-HoVO_4_ NPs (144 mg Ho/kg) or iohexol (112 mg I/kg).

**Figure 11:**
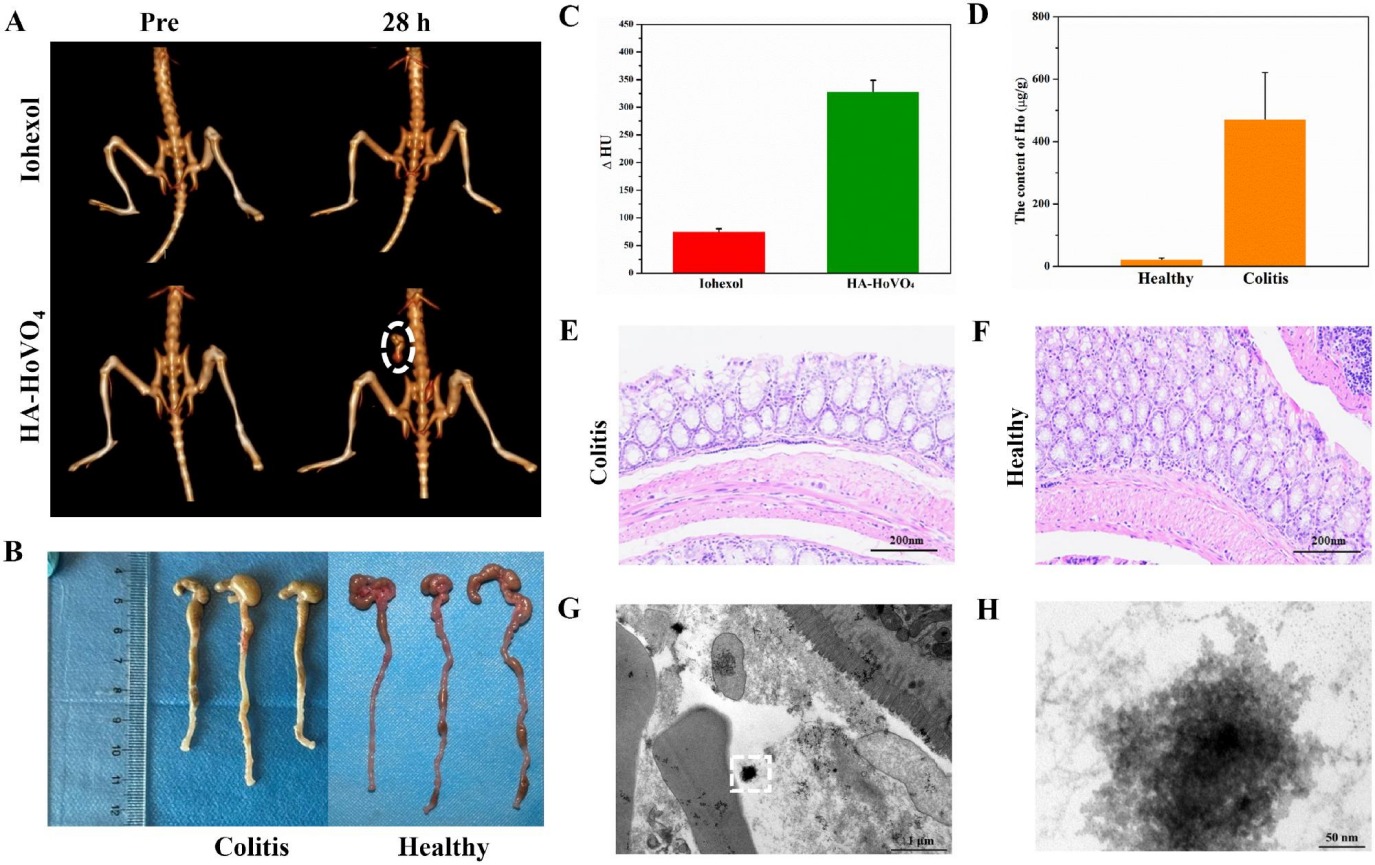
Accumulation of HA-HoVO_4_ NPs in the inflamed colon of DSS-induced colitis mice. (**A**) Representative CT images of colitis mice acquired pre and at 28 h after oral administration of iohexol (112 mg I/kg) or HA-HoVO_4_ NPs (144 mg Ho/kg). The white dashed ellipse highlighted specific nanoparticle accumulation in inflamed colon regions. (**B**) Photos of colon tissues from colitis and healthy mice. (**C**) CT attenuation values measured in the colon of colitis mice 28 h after administration of HA-HoVO_4_ or iohexol (*n* = 3). (**D**) Ho content in colon tissues of healthy and colitis mice 28 h after HA-HoVO_4_ administration, quantified by ICP-OES (*n* = 3). H&E-stained colon sections from (**E**) colitis and (**F**) healthy mice, scale bar: 100 μm. (**G**) Representative TEM image showing HA-HoVO_4_ NPs accumulation in the colon of colitis mice at 28 h. (**H**) Enlarged view of the area within the white box in (**G**).

To assess the performance of HA-HoVO_4_ NPs for colitis imaging, the acute colitis model was established by administering 5% DSS in drinking water for 6 days (Figure 10A). Mice in the DSS-treated group exhibited significant weight loss (Figure S12), and most of the mice showed mucoid stools, and some presented bloody stools. The DAI scores of the DSS model group gradually increased over time, which was consistent with the trend of histopathological changes (Figure S13). Next, *in vivo* CT imaging was performed in DSS-induced colitis mice and healthy mice following oral administration of HA-HoVO_4_ NPs or iohexol. Within 4 h after administration of 200 μL of HA-HoVO_4_ NPs or iohexol, gastrointestinal transit patterns were similar in colitis and healthy mice, and gastrointestinal outlines were visible for both groups. However, iohexol provided weak contrast in the stomach and was rapidly cleared from the intestines. At 28 h post-administration, iohexol was almost completely cleared from the gastrointestinal tract, whereas HA-HoVO_4_ NPs showed significant accumulation in the inflamed colon regions (Figures 10B and 11A), demonstrating their selective retention in diseased tissue. In the barium sulfate-treated group, nearly all the barium sulfate was excreted from the body within 12 h, and no residual signal was detected at the inflamed site in the large intestine at 24 h (Figure S14). These results preliminarily demonstrated the specific targeting ability of HA-HoVO_4_ NPs toward inflammatory lesions.

Then, colon length and tissue morphology were analyzed across all mice, and DSS-treated mice exhibited significantly shorter colon lengths compared to the control group (Figure 11B). In addition, a higher CT value was observed in the inflamed large intestine of DSS-colitis mice administered HA-HoVO_4_ NPs compared to those receiving iohexol (Figure 11C). Quantitative analysis by ICP-OES further demonstrated a greater accumulation of Ho in the colon tissue of colitis mice relative to healthy controls 28 h after HA-HoVO_4_ NPs administration (Figure 11D). The Ho element content of HA-HoVO_4_ NPs in the colon tissue of colitis mice was also markedly higher than that of HoVO_4_ NPs (Figure S15). These findings suggested the inflammation-specific targeting capability of the nanoparticles.

Moreover, histopathological examination of colonic sections (Figure 11E and F) further confirmed the successful modeling, in which colitis mice exhibited disrupted intestinal structure and inflammatory infiltration, whereas intact colonic mucosa was observed in healthy controls. As shown in Figure 11G and H, TEM images visually confirmed the specific accumulation of HA-HoVO_4_ NPs within colonic mucosal epithelial cells and intercellular spaces of colitis mice at 28 h post-administration. These findings underscore the feasibility and potential of HA-HoVO_4_ NPs for targeted CT imaging of colitis, providing a reliable approach for monitoring inflammatory bowel diseases. These results show that HA-HoVO_4_ NPs have demonstrated higher sensitivity than conventional CT imaging for the diagnosis of digestive diseases.

**Figure 12:**
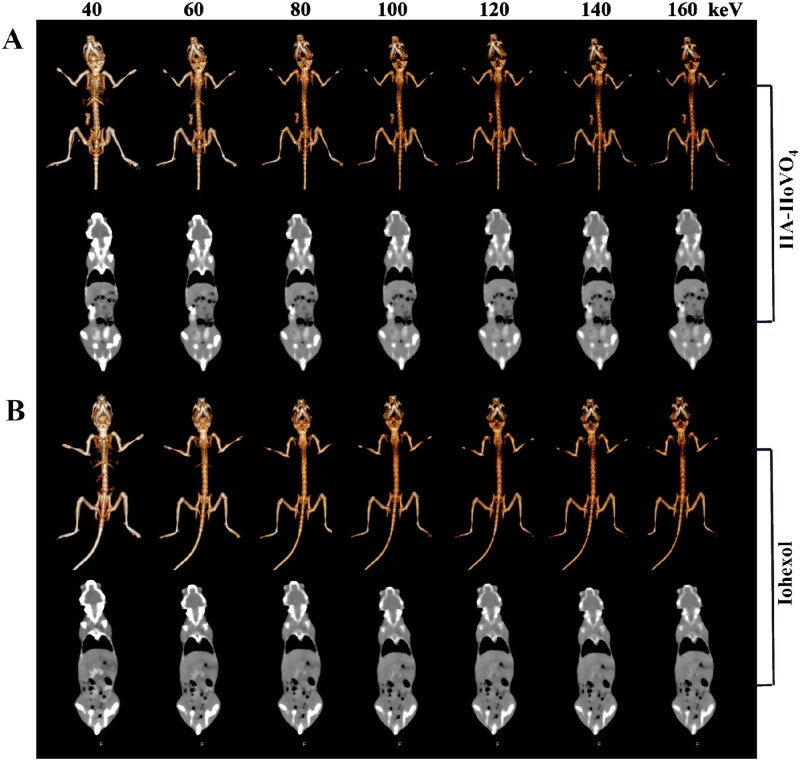
*In vivo* spectral CT imaging of the gastrointestinal tract in colitis mice at 28 h after oral administration of 200 μL HA-HoVO_4_ NPs (144 mg Ho/kg) or iohexol (112 mg I/kg), acquired at different monochromatic energies. (**A**) 3D reconstruction and coronal CT images of colitis mice treated with HA-HoVO_4_ NPs. (**B**) 3D reconstruction and coronal CT images of colitis mice treated with iohexol.

*In vivo* spectral CT imaging was conducted in colitis mice at 28 h after oral administration of 200 μL of HA-HoVO_4_ NPs or iohexol. 3D reconstruction and coronal CT images were acquired at multiple monochromatic energy levels (40, 60, 80, 100, 120, 140 and 180 keV) using a spectral CT workstation (Figure 12). As the monochromatic energy increased from 80 to 160 keV, X-ray attenuation in surrounding tissues decreased significantly, while the CT signal from HA-HoVO_4_ NPs accumulated in colitis regions remained detectable and relatively stable, which enabled high-contrast visualization of lesions across a broad energy range (Figure 12A). In contrast, iohexol produced only weak CT signals in the gastrointestinal tract of colitis mice even at 40 keV, and its signal became negligible at monochromatic energies above 60 keV (Figure 12B). To compare the imaging performance of HA-HoVO_4_ NPs and iohexol at different energy levels, ROIs were defined in the target tissue (inflammatory site) and adjacent muscle (background). The results (Figure S16) showed that the CNR value of HA-HoVO_4_ NPs was significantly higher than that of iohexol. These results suggested that HA-HoVO_4_ NPs possessed distinct advantages over conventional CT contrast agents in the detection of colitis using spectral CT imaging.

## Discussion

The development of contrast agents for gastrointestinal spectral CT imaging has seen considerable progress, yet existing candidates still present several limitations. For example, bismuth oxide (Bi_2_O_3_) nanoparticles possess a high atomic number and strong X-ray attenuation but suffer from inconsistent particle size distribution and poor reproducibility [6, 49]. Dextran-coated cerium nanoparticles offer good biocompatibility and active targeting toward inflamed GI tissue; however, the relatively low K-edge energy of cerium results in insufficient energy-dependent attenuation, limiting their efficacy in multi-energy spectral CT [9]. Conventional iodine-based agents are constrained by a lower atomic number compared to rare earth elements, leading to suboptimal contrast enhancement in spectral CT imaging [21]. These situations underscore the need for novel GI contrast agents that combine structural stability with favorable X-ray attenuation properties. The lanthanide element Ho presents a compelling candidate due to its high atomic number, abundant sub-shell electrons, and strong X-ray attenuation capacity. To highlight the advantages of our system, we compared HA-HoVO_4_ NPs with representative contrast agents, including PEGylated NaHoF_4_, Bi_2_O_3_ NPs, and iodinated contrast agents (e.g. iohexol), based on the following key parameters: X-ray attenuation coefficient (or mass attenuation coefficient at relevant energies), K-edge energy, biodistribution profile, and reported toxicity (Supplementary Table S1). Despite these advantages, no Ho-based nanoparticles have been reported to date for GI spectral CT imaging, which represents a significant opportunity for material innovation and diagnostic application.

In this work, HA-HoVO_4_ nanoparticles developed features straight forward synthesis, high yield, excellent water solubility, and favorable biocompatibility. Moreover, high cell viability in RAW264.7, MCF-10A and CT26 cell lines, even at a concentration of 200 μg/mL, indicates that HA-HoVO_4_ nanoparticles have relatively low cytotoxicity. Targeted cellular uptake confirmed their specific binding capability toward inflammatory cells via the interaction of the HA unit and CD44 receptor. HA-HoVO_4_ NPs exhibited superior CT performance and enhanced contrast compared to conventional iodinated agents both *in vitro* and *in viv*o. *In vitro* imaging showed higher CT values in the HA-HoVO_4_ NPs group at equivalent elemental concentrations, and oral administration of HA-HoVO_4_ NPs led to clearer delineated gastrointestinal tract structures with improved contrast. In rat models of acute gastritis induced by alcohol and aspirin, HA-HoVO_4_ NPs accumulated more extensively in the gastric wall than iohexol. In addition, TEM imaging and ICP-OES analysis verified their specific enrichment within inflamed gastric tissues, indicating that HA-HoVO_4_ NPs achieved higher sensitivity and diagnostic accuracy in spectral CT imaging for gastritis. Similarly, in acute colitis models, HA-HoVO_4_ NPs selectively accumulated at sites of intestinal inflammation, demonstrating clear potential for inflammatory bowel disease diagnosis. Collectively, these findings highlighted HA-HoVO_4_ NPs as a promising contrast agent for spectral CT imaging of gastrointestinal diseases.

Nevertheless, this study has several limitations that should be acknowledged. The biosafety assessment remains preliminary, although no adverse effects were observed under the experimental conditions, the evaluation was limited to short-term observations in healthy animals. Further investigation is necessary to assess potential long-term accumulation, chronic toxicity and the metabolic fate of HA-HoVO_4_ NPs, particularly following repeated administrations. In addition, the animal models used were restricted to rodents (mice and rats). While these models are valuable for initial proof-of-concept studies, they may not fully replicate the complexity of gastrointestinal inflammation or the physiological responses in larger animals or humans.

Regarding the ethanol/aspirin-induced acute gastritis model, the establishment method followed a previously reported protocol [4]. Model validation was performed solely by H&E staining to observe gastric histopathological changes (e.g. mucosal erosion, inflammatory cell infiltration). However, no systematic severity scoring system or additional validation methods were employed. This limitation reflects our initial focus on evaluating the targeting performance of the nanoparticles rather than comprehensive model characterization. In future studies, we plan to adopt a more rigorous evaluation system, including histopathological scoring and detection of inflammation-related indicators, to provide more robust data support. Furthermore, the oral dose of 144 mg Ho/kg (approximately 0.87 mmol Ho/kg) used in this study was selected based on optimal imaging efficacy in murine models of gastrointestinal inflammation. Although this molar dose falls within the same order of magnitude as clinical iodine-based contrast agents (0.6–1.2 mmol I/kg for adults) [50, 51], direct dose extrapolation to humans remains uncertain and warrants careful validation in future clinical trials.

## Conclusion

In conclusion, we successfully synthesized HA-HoVO_4_ NPs and systematically evaluated their performance as a spectral CT contrast agent for gastrointestinal imaging. The results demonstrate that HA-HoVO_4_ NPs possessed excellent biocompatibility, minimal cytotoxicity, and superior X-ray attenuation properties compared to traditional iodine-based contrast agents. Both *in vitro* and *in vivo* experiments confirmed that HA-HoVO_4_ NPs enable clear imaging at low concentrations. In murine models of gastritis and colitis, oral administration of HA-HoVO_4_ NPs allowed precise visualization of inflammatory lesions, which specifically accumulated at disease sites and further improved diagnostic accuracy. These findings support HA-HoVO_4_ NPs as a highly promising and biosafe spectral CT contrast agent, offering a viable strategy for non-invasive diagnosis and monitoring of gastrointestinal inflammatory diseases.

## Supplementary Material

rbag106_Supplementary_Data
