# Supplementary material for: Holmium vanadate nanoparticles as a biosafe and efficient contrast agent for spectral CT imaging of gastritis and colitis
Source: Regen Biomater. 2026 Jun 2;13:rbag106. doi: 10.1093/rb/rbag106 (PMC13294444; doi:10.1093/rb/rbag106)
Supplement: rbag106_Supplementary_Data [file rbag106_supplementary_data.docx]

**Supporting Information**

**Holmium vanadate nanoparticles as a biosafe and efficient contrast agent for spectral CT imaging of gastritis and colitis**

Wenqian Ru^a, b†^, Chunmei Yang^b†^, Xin Zhu^b†^, Yanlin Wu^a^, Fei Pei^a^, Haoran Chen^a^, Yuanyuan Ma^a^, Qiuyu Meng^c*^, Lu Yang^a*^, Yong Xu^a*^

*^a^ Department of Endocrinology and Metabolism, The Affiliated Hospital of Southwest Medical University, Sichuan Clinical Research Center for Nephropathy, Metabolic Vascular Disease Key Laboratory of Sichuan Province, and Sichuan-Chongqing Joint Key Laboratory of Metabolic Vascular Diseases, Luzhou, Sichuan 646000, China*

*^b^ Department of Radiology, The Affiliated Hospital, Southwest Medical University, Precision Imaging and Intelligent Analysis Key Laboratory of Luzhou, Luzhou, Sichuan 646000, China*

*^c^ Zhejiang Collaborative Innovation Center for Full-Process Monitoring and Green Governance of Emerging Contaminants，Interdisciplinary Research Academy (IRA), Zhejiang Shuren University, Hangzhou 310015, China*

*^†^These three authors contributed equally to this work*

**Correspondence to: Qiuyu Meng,* [*qiuyumeng@zjsru.edu.cn*](mailto:qiuyumeng@zjsru.edu.cn)*; Lu Yang,* [*yanglu@swmu.edu.cn*](mailto:yanglu@swmu.edu.cn)*; Yong Xu, xywyll@swmu.edu.cn*

**Supplementary Table and Figures**


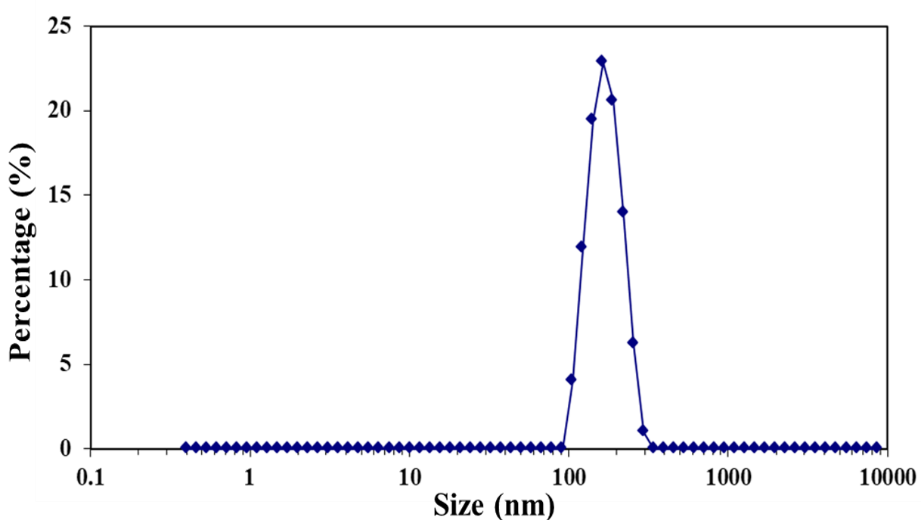


**Figure S1** Hydrodynamic size of HA-HoVO_4_ NPs.


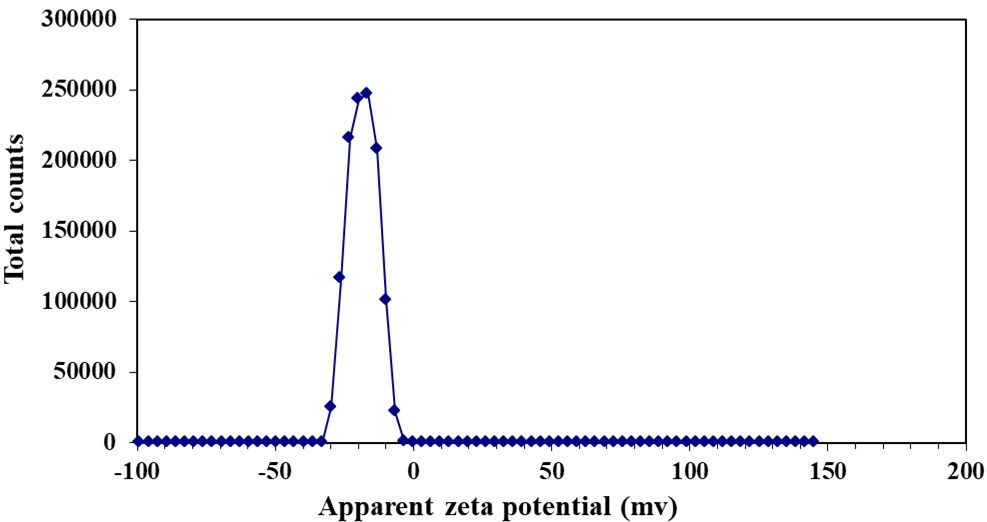


**Figure S2** Apparent zeta potential of HA-HoVO_4_ NPs.


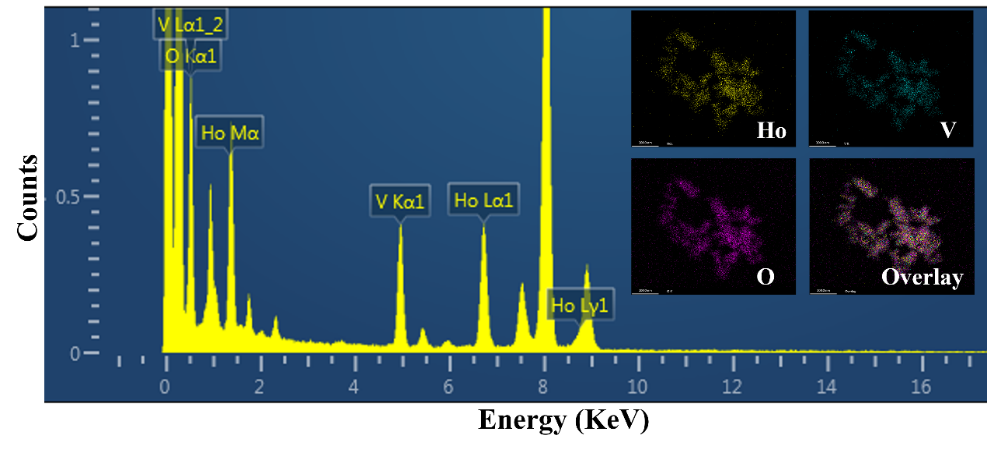


**Figure S3** Energy dispersive spectroscopy (EDS) spectrum of HA-HoVO_4_ NPs.


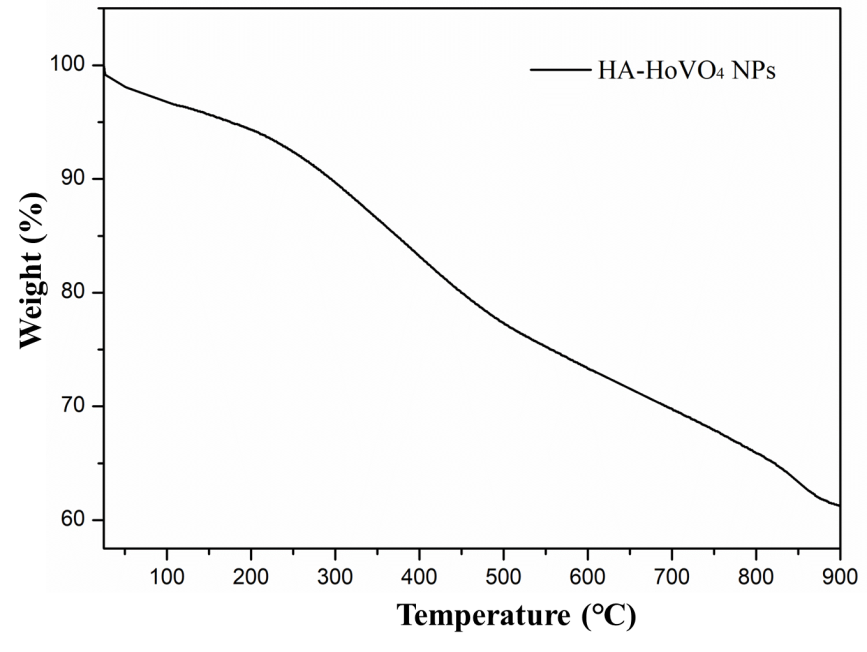


**Figure S4** TGA curves of HA-HoVO_4_ NPs.


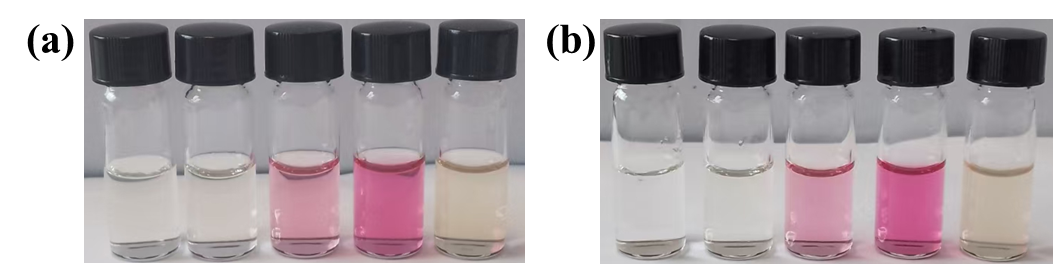


**Figure S5** Colloidal stability of HA-HoVO_4_ NPs in various physiological media. Photographs of HA-HoVO_4_ NPs dispersions in (from left to right) 0.9% NaCl, PBS, RPMI-1640, DMEM, and FBS after incubation at 37°C for (a) 7 days and (b) 14 days.


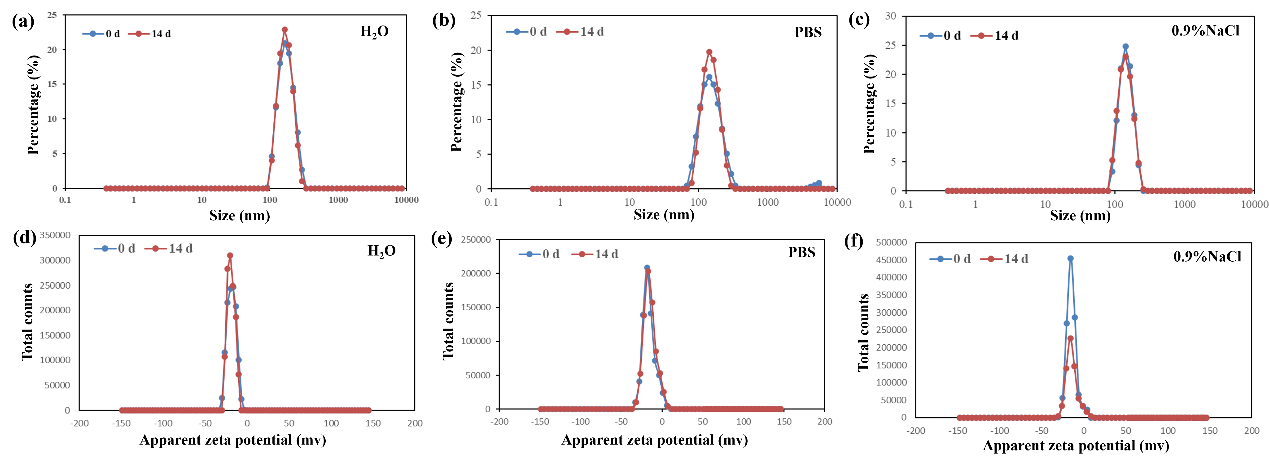
**Figure S6** Hydrodynamic size and apparent zeta potential over time of HA-HoVO_4_ NPs dispersed in different media, including (a, d) H_2_O, (b, e) PBS, and (c, f) 0.9% NaCl solution.


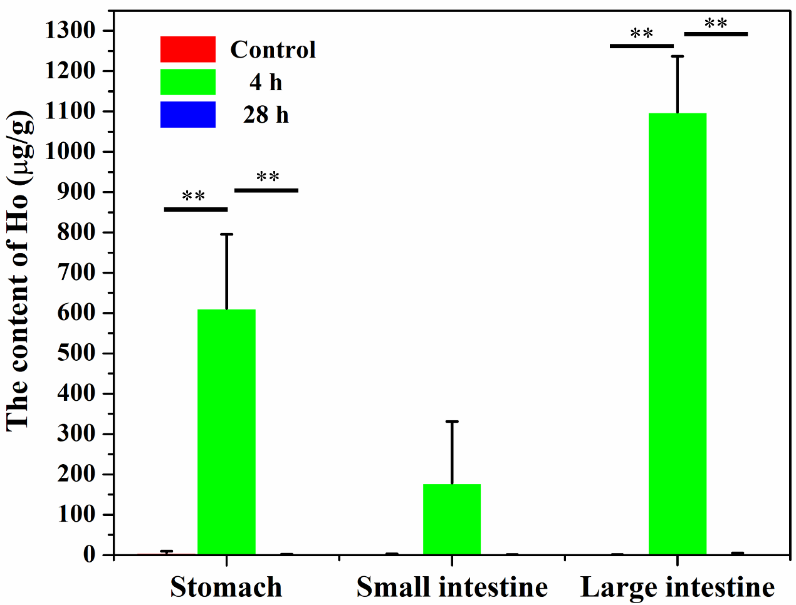


**Figure S7** Time-dependent distribution of HA-HoVO_4_ NPs in gastrointestinal tract (stomach, small intestine, and colon) at 0 h, 4 h, and 28 h after gavage. ***p* < 0.01.


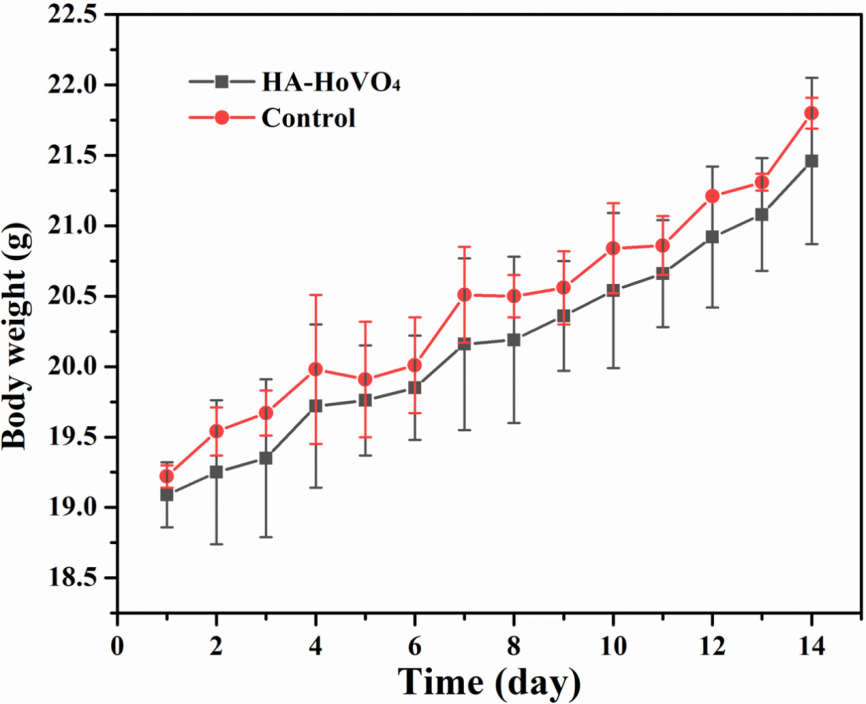


**Figure S8** Daily body weight changes of mice in each experimental group.


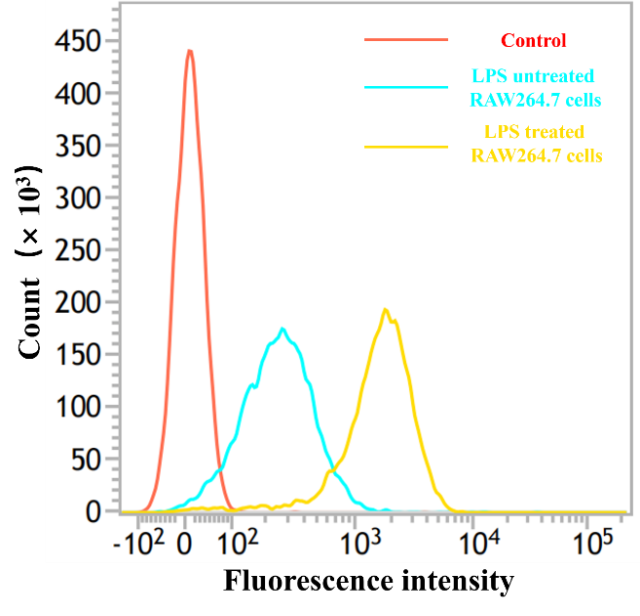


**Figure S9** Flow cytometry analysis of CD44 expression in LPS treated and untreated RAW264.7 cells. Cells were stained with CD44-labeled Alexa Fluor®488 antibody.


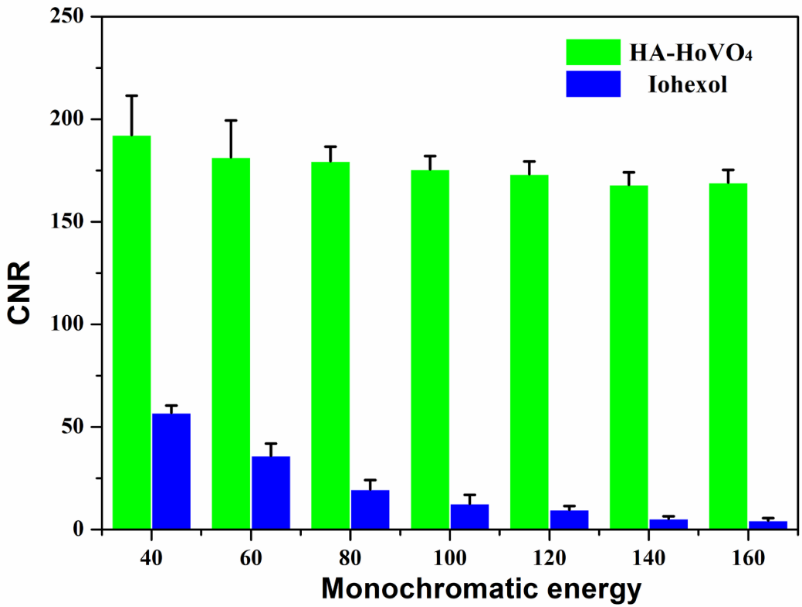


**Figure S10** Contrast-to-Noise Ratio (CNR) of ROI in normal mice after treating with HA-HoVO_4_ NPs or iohexol for 2 h.


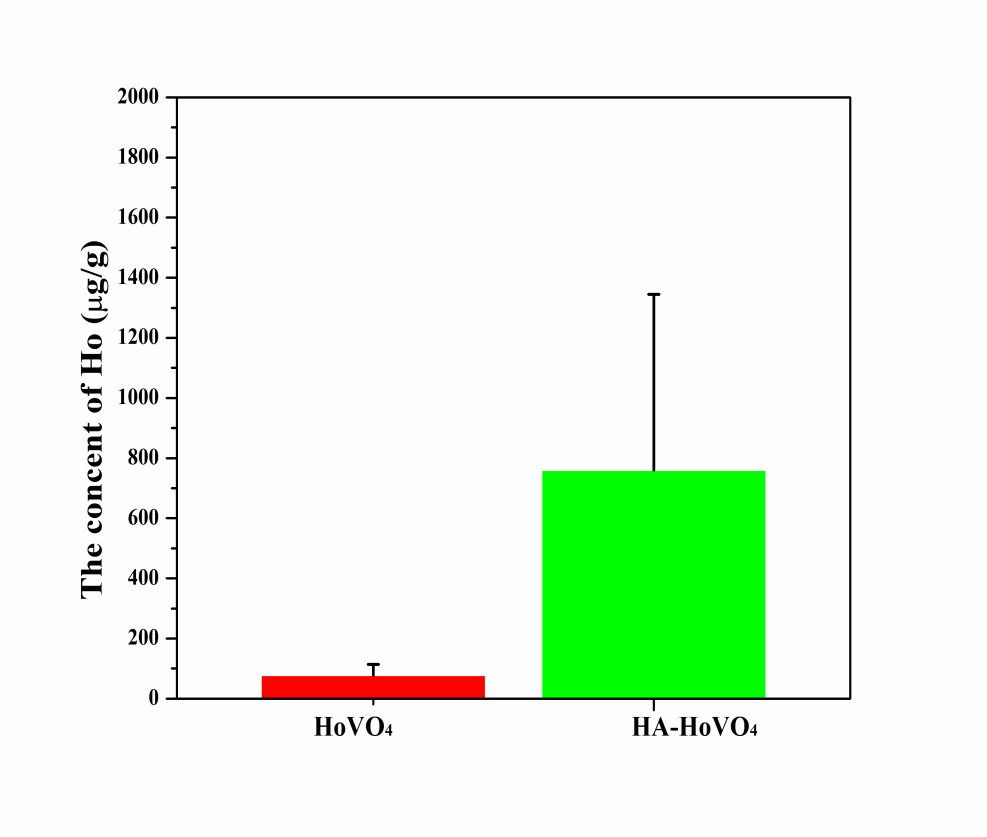


**Figure S11** Ho content in stomach tissues of gastritis rats 24 h after HoVO_4_ and HA-HoVO_4_ NPs administration, quantified by ICP-OES.


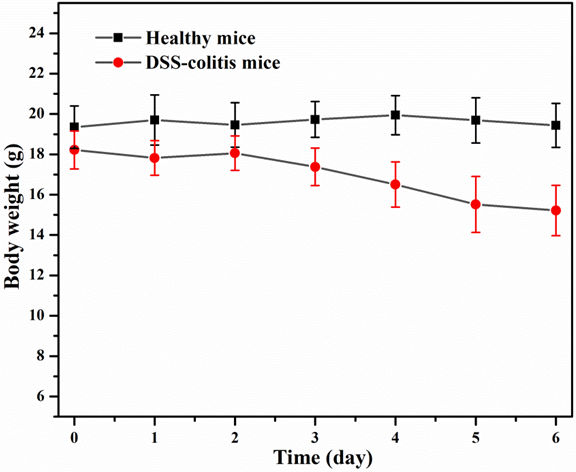


**Figure S12** Body weight changes over time in healthy and colitis mice.


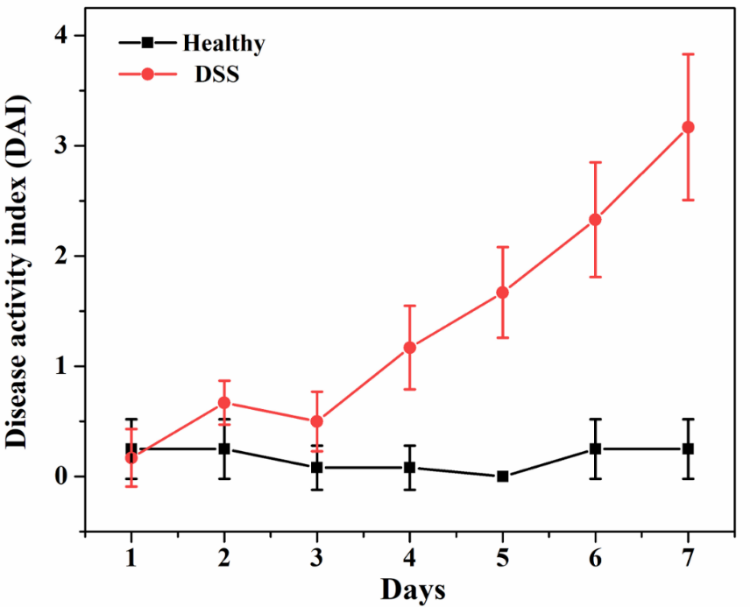


**Figure S13** Daily variations in Disease Activity Index (DAI) scores across different treatment groups in mice.

**
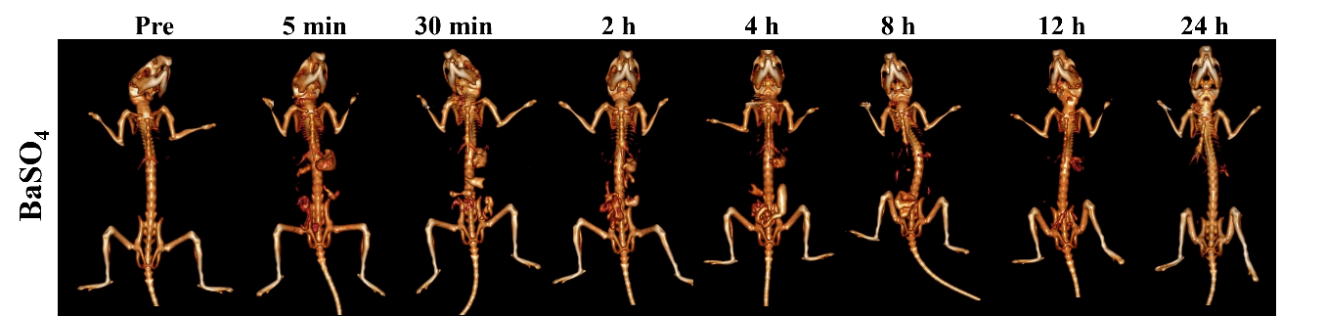
**

**Figure S14** In vivo CT imaging of the gastrointestinal tract in colitis mice after oral administration of 200 μL BaSO_4_ suspension (120 mg of Ba/kg body weight).


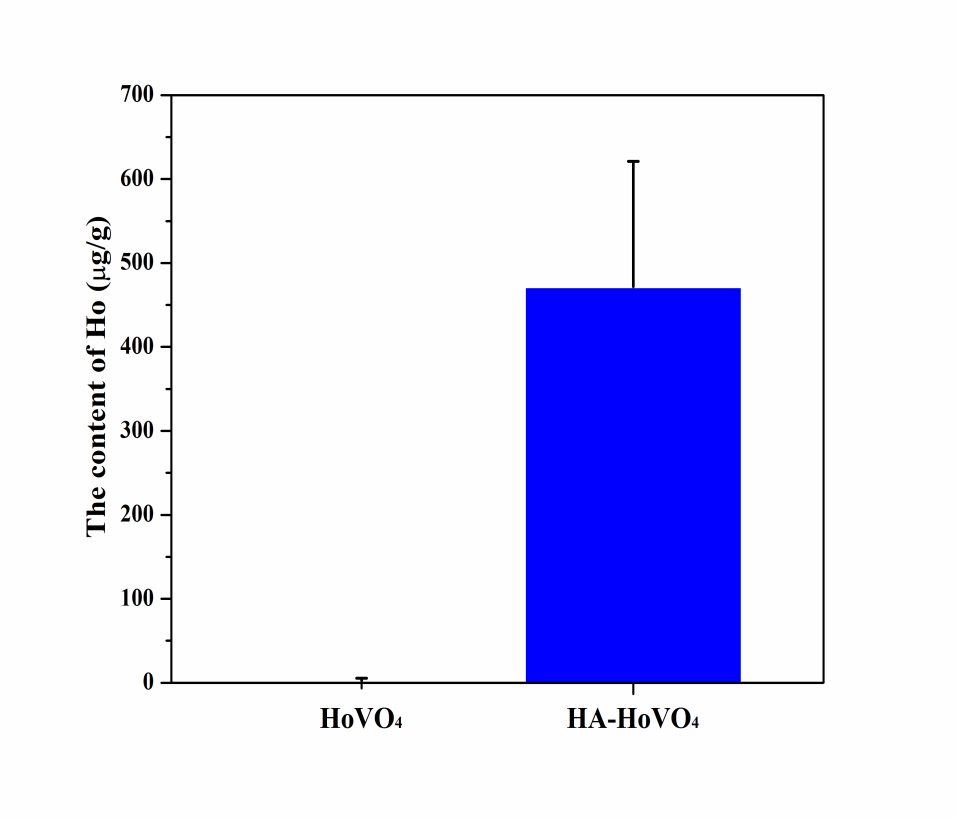


**Figure S15** Ho content in colon tissues of colitis mice 28 h after HoVO_4_ and HA-HoVO_4_ NPs administration, quantified by ICP-OES.


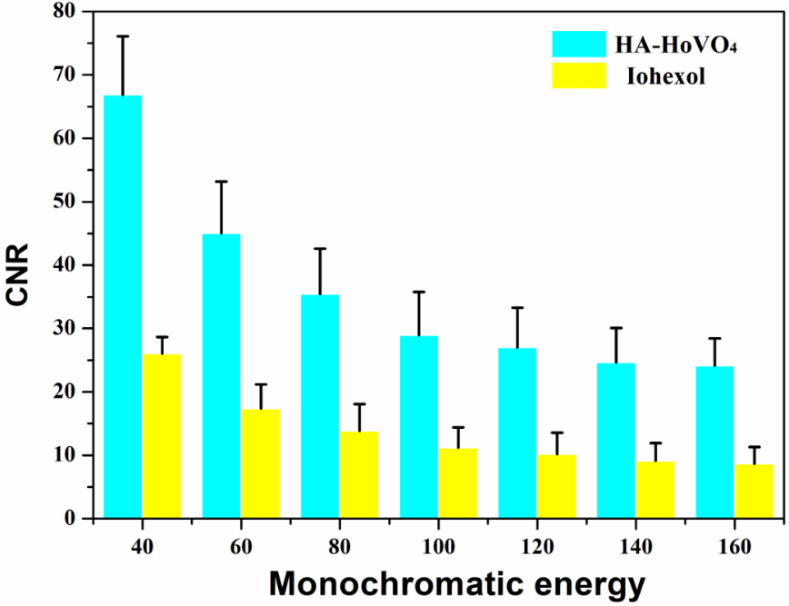


**Figure S16** Contrast-to-Noise Ratio (CNR) of ROI in colitis mice after treating HA-HoVO_4_ NPs or iohexol for 24 h.

**Table S1** Comparison of key parameters between HA-HoVO_4_ NPs and representative X-ray imaging contrast agents.

|  | HA-HoVO_4_ | Bi_2_O_3_ | PEG-NaHoF_4_ | I |
| --- | --- | --- | --- | --- |
| X-ray attenuation coefficient(cm^2^/g, 100 keV） | 3.49 (Ho) | 5.74 (Bi) | 3.49 (Ho) | 1.94 (I) |
| K-edge energy (keV) | 53.8 (Ho) | 90.5 (Bi) | 53.8 (Ho) | 33.2 (I) |
| Biodistribution | Hepatic clearance | Accumulation in liver and spleen | Predominantly enriched in liver and spleen | Renal clearance |
| Toxicity profile | Low toxicity | Potential heavy metal toxicity | Low toxicity; prolonged in vivo retention | Risk of nephrotoxicity at high doses |
